# Microbiota and Metabolite Modifications after Dietary Exclusion of Dairy Products and Reduced Consumption of Fermented Food in Young and Older Men

**DOI:** 10.3390/nu13061905

**Published:** 2021-06-01

**Authors:** Jinyoung Kim, Kathryn J. Burton-Pimentel, Charlotte Fleuti, Carola Blaser, Valentin Scherz, René Badertscher, Corinne Marmonier, Noëlle Lyon-Belgy, Aurélie Caille, Véronique Pidou, Adeline Blot, Claire Bertelli, Jérémie David, Ueli Bütikofer, Gilbert Greub, Dominique Dardevet, Sergio Polakof, Guy Vergères

**Affiliations:** 1 Unité de Nutrition Humaine (UNH), INRAE, Université Clermont Auvergne, 63000 Clermont-Ferrand, France; jinyoung.kim@inrae.fr (J.K.); jeremie.david@inrae.fr (J.D.); dominique.dardevet@inrae.fr (D.D.); sergio.polakof@inrae.fr (S.P.); 2 Agroscope, Federal Department of Economic Affairs, Education and Research EAER, CH-3003 Bern, Switzerland; charlotte.fleuti@agroscope.admin.ch (C.F.); carola.blaser@agroscope.admin.ch (C.B.); rene.badertscher@agroscope.admin.ch (R.B.); ueli.buetikofer@agroscope.admin.ch (U.B.); guy.vergeres@agroscope.admin.ch (G.V.); 3 CNIEL, 42 Rue de Châteaudun, F-75009 Paris, France; cmarmonier@cniel.com; 4 Institute of Microbiology, Lausanne University Hospital and Lausanne University, CH-1011 Lausanne, Switzerland; Valentin.Scherz@chuv.ch (V.S.); Claire.Bertelli@chuv.ch (C.B.); gilbert.greub@chuv.ch (G.G.); 5 Centre Hospitalier Universitaire (CHU) Clermont Ferrand, CRNH Auvergne, F-63000 Clermont-Ferrand, France; nlyonbelgy@chu-clermontferrand.fr (N.L.-B.); acaille@chu-clermontferrand.fr (A.C.); vpidou@chu-clermontferrand.fr (V.P.); ablot@chu-clermontferrand.fr (A.B.)

**Keywords:** dairy products, fermented food, gut microbiota, ageing, lipids/free fatty acids

## Abstract

The gut microbiota adapts to age-related changes in host physiology but is also affected by environmental stimuli, like diet. As a source of both pre- and probiotics, dairy and fermented foods modulate the gut microbiota composition, which makes them interesting food groups to use for the investigation of interactions between diet and ageing. Here we present the effects of excluding dairy products and limiting fermented food consumption for 19 days on gut microbiota composition and circulating metabolites of 28 healthy, young (YA) and older (OA) adult men. The intervention affected gut microbial composition in both groups, with significant increases in *Akkermansia muciniphila* and decreases in bacteria of the Clostridiales order. Lower fasting levels of glucose and insulin, as well as dairy-associated metabolites like lactose and pentadecanoic acid, were observed after the intervention, with no effect of age. The intervention also decreased HDL and LDL cholesterol levels. Dairy fat intake was positively associated with the HDL cholesterol changes but not with the LDL/HDL ratio. In conclusion, restricting the intake of dairy and fermented foods in men modified their gut microbiota and blood metabolites, while the impact of the dietary restrictions on these outcomes was more marked than the effect of age.

## 1. Introduction

The last two decades have revealed a close relationship between the gut microbiota and human health. Indeed, the gut microbiota composition has been associated with many diseases and metabolic dysfunction, including obesity, inflammation, metabolic syndrome, intestinal bowel diseases, and cancer [1]. Among the potential determinants of the gut microbiota composition, environmental factors, including diet, account for over 20% of inter-individual β-diversity, a profound effect that surpasses the contribution of host genetics [2]. Diet is a modifiable modulating factor of the gut microbiota composition with numerous studies having demonstrated the impact of different dietary patterns including fibre-rich diets [3], Western diet [4], and Mediterranean diet [5], on the gut microbiota composition. These changes were accompanied by biological changes in the host that reflect in part the metabolic processing of the ingested nutrients by microorganisms in the gut. Often dietary modulations of the gut microbiota have been linked to the presence of pre- or probiotics that are widely present in the diet, in particular in fermented foods including yogurt, bread, pickles, and kimchi [6,7].

Dairy products comprise an important food group that has been widely investigated for its role on the gut microbiota composition [8,9]. The probiotic bacteria present in fermented dairy products include not only well-studied species, such as *Bifidobacteria* spp., that are added independently of the fermentation procedure, but also other lactic acid bacteria (LAB) that are frequently used as starter cultures in fermented dairy production. Oligosaccharides, an important prebiotic group that influence the gut microbiota composition, are also present in dairy products [10,11]. Though not classified as a prebiotic, lactose can reach the colon, particularly in individuals with low lactase activity, where it is an energy substrate for the gut microorganisms exerting effects similar to other indigestible carbohydrates [12]. Fermented dairy products also contain various bacterial end-products, which act as signalling molecules in the gut microbiota community, but also influence host fitness by other mechanisms [13]. For example, indoles (that are products of microbial tryptophan metabolism [6]) stimulate host intestinal mucin production, thereby providing an energy source to commensal bacteria and protecting the host intestinal layer from pathogen invasion [14]. Both LAB and metabolites derived from the fermentation process in dairy products thus have the potential to modulate the gut microbiota composition and mediate some of the health benefits of dairy products [15,16,17,18]. However, these effects are dependent on multiple factors including the metabolite flux across the digestive tract after dairy product consumption, the host microbiota composition, and the metabolic or health status of the consumer.

Dairy products are promoted for their nutritional properties across the lifespan, from children [19] to elderly populations [20]. The different ways by which they might support healthy ageing are still under investigation and subject to some debate [20,21]. The ageing process is associated with many changes in physiology and lifestyle parameters, including decreased intestinal motility, altered taste and smell perception, impaired dentition and reduced chewing strength, altered dynamics of nutrient turnover and impaired ability to maintain energy homeostasis [22]. These changes can lead to difficulties in eating, altering nutrient intake for both the host and gut microbiota, ultimately influencing the composition of the latter as the organism ages [23]. Indeed, distinct characteristics of the gut microbiota, in particular its composition, have been identified in older populations in comparison to younger populations [24]. The changes of the gut microbiota composition in older populations modifies its functional and transcriptional activities, which, in consequence, might affect host metabolism [25,26]. Recently, the ‘NU-AGE’ study demonstrated that a modification of the gut microbiota composition in frail older individuals following the Mediterranean diet intake for one year was accompanied by a reduction in the low-grade inflammation, typically associated with ageing (‘inflammaging’) [5]. This supports a role for dietary interventions to promote healthy ageing via a potential intermediary effect on the gut microbiota composition and functionality.

While both diet and ageing appear to be major factors that modulate the gut microbiota composition and host metabolism, their interactions remain unclear. To investigate the effects of age on diet-microbiota interactions, we report here on the microbiota and circulating metabolite changes of healthy young and older men who, in the context of a further nutritional intervention study investigating milk and yoghurt consumption, underwent a 19 day period of complete exclusion of dairy products and restriction in major dietary sources of probiotics (fermented foods). We aimed to disentangle the independent effects of age and diet on microbiota and metabolite outcomes from the effects of age as a mediator of the response to diet.

## 2. Materials and Methods

### 2.1. Study Design

**Figure 1:**
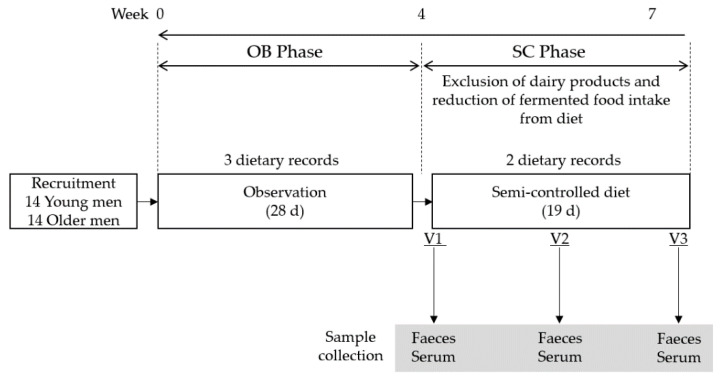
Overview of study design. A total of 14 healthy young adult men (YA) and 14 older adult men (OA) participated in a 28-day observation phase (OB phase) with 3 dietary records conducted at intervals of 9 ± 4 days. The semi-controlled diet phase (SC phase) excluded dairy product intake and reduced fermented food intake for 19 days in a free-living condition. During the SC phase, fasting serum samples were collected on three visits (V1 on day 1, V2 on day 8–14, V3 on day 19 of intervention). Faecal samples were collected either a day before each visit or on the morning of the visit.

The current report uses data obtained from a larger study in which a 19-day controlled diet intervention (semi-controlled diet (SC) phase) was set up to remove all dairy products from the diet. As fermented foods could confound the effects of dairy products, the participants were also instructed to reduce their consumption of these foods (Figure 1). A list of the foods allowed for consumption, as well as those prohibited (Table S1), were provided to the participants, together with dietary advice for implementing these restrictions. Fermented foods were defined according to the classification strategy used by Li, et al. [27]. To assess the adherence of the participants to the dietary restrictions, two dietary records were collected at the end of the first and second week of the SC phase. Participants visited the research centre three times during the SC phase (V1: d1, V2: d8-d14, V3: d19) and at each visit sera was collected in the morning after an overnight fast. Faecal samples were collected either a day before the visit or on the morning of the visit; in the former case, the participants kept their sample in a conventional freezer until the following morning. A 28-day observation phase (OB phase) preceded the SC phase. During the OB phase, the participants were asked to maintain their habitual diet, which was assessed by three 3-day food records conducted at intervals of 5–10 days from the beginning to the end of the OB phase. Body weight was recorded at V1 and V3.

### 2.2. Study Population

A total of 28 healthy men participated in the study, fourteen young adult men (YA) and fourteen older adult men (OA). However, one OA was excluded due to use of antibiotics during the intervention, thus all statistical analysis was performed using thirteen OA (median age of 69.0 y (Q1:66.0, Q3: 71.0)) and fourteen YA (median age of 27.5 y (Q1: 25.0, Q3: 31.0)) (Figure S1). The choice to study men in the context of a study on metabolic differences in older and younger subjects was motivated by the potential confounding of age differences by hormonal differences in women pre- and post-menopause. In addition, this selection criterion aimed to limit the inter-individual variation of the population.

Participants were screened by a telephone interview and, for those eligible, invited to a medical visit. During this screening visit, health status, anthropometrics, and selection criteria were assessed. Inclusion criteria were absence of chronic or acute illness, aged from 20 to 35 years for the YA group or 65 to 80 years for the OA group, BMI from 21 to 30 kg/m^2^, regular intake of dairy products (>2 portions per day). Exclusion criteria were defined as smoking > 5 cigarettes per day, alcohol intake > 3 glasses per day, sport activity > 6 h per week, diagnosed health conditions (chronic or acute disease), regular use of medication, regular intake of nutritional supplements, antibiotics treatment within 1 month of study enrolment, food allergies or intolerance, special diets (vegetarians, non-consumers of dairy products), medical history of anaemia, unwillingness to follow dietary restrictions or the study controlled diet, participation in a different clinical study at the same time, and blood donation within three months prior to study enrolment. All participants provided informed written consent during enrolment. The trial was approved by the Ethical Committee of Personal Protection (CPP Ile de France IV) and legal authorities and was registered in April 2018 at www.clinicaltrials.gov accessed on 1 March 2021 (NCT03500003). All study visits were conducted at the Human Nutrition Research Centre of Auvergne (CRNH-A; Clermont-Ferrand, France) according to French law between July 2018 and March 2019. All procedures were carried out in accordance with the guidelines laid down in the Declaration of Helsinki.

### 2.3. Dietary Assessment

Participants recorded their food consumption five times using 3-day food records (each comprising two weekdays and one weekend day). The first three assessments were completed during the OB phase and the last two during the SC phase. Dietary records were analysed by a dietitian using NUTRILOG^®^ (version 3.21b) with the food composition table CIQUAL^®^ (2017, https://ciqual.anses.fr/, accessed on 1 September 2019), and dietary portions album SuViMax^®^ (Polytechnica Edition) to validate the portions of food consumed (in grams). The raw dietary data were extracted as 93 food subgroups (33 food groups) and 30 nutrients categories (CIQUAL^®^, 2017). For the purpose of this study, the food subgroups were then re-categorised into 58 food subgroups forming eleven food groups based on the updated CIQUAL^®^ table (2020) (https://ciqual.anses.fr/, accessed on 1 October 2020) as well as by the characteristics of the design of current study (Table S2). In addition, according to the strategy developed by Li, et al. [27], foods were classified into fermented foods and non-fermented foods, with subsets of dairy: fermented dairy foods and non-fermented dairy foods. The dietary analyses were conducted using the mean daily food group and food subgroup intakes, macro- and micronutrient intakes, and the proportion of energy intake from different macronutrient sources. Food groups and subgroups that were only consumed in <25% of the population for both OB and SC phases were excluded from statistical analyses.

### 2.4. Serum Analyses

#### 2.4.1. Biochemical Analyses

Fasting serum glucose, cholesterol (total, HDL, LDL), triglycerides, urea, and lactate levels were measured by spectrophotometry on an automated chemistry analyser (ABX Pentra 400, Horiba, Montpellier, France) using kits from ABX Pentra. The concentrations of insulin were measured by a commercially available enzyme-linked immunosorbent assay (ELISA) kit (Mercodia SAS, Paris, France). The inflammation markers C- reactive protein (CRP) and adiponectin were also quantified using commercially available enzyme-linked immunosorbent assay (ELISA) kits (CRP: Human hs-CRP ELISA Kit, Elabscience Biotechnology Co. Nanterre, France; Adiponectine: Invitrogen™ Kit ELISA, Fisher Scientific SAS, llkirch, France) according to the manufacturers’ instructions. Non esterified fatty acids (NEFA) were measured by an in vitro enzymatic colorimetric method assay (HR series NEFA-HR (2), Fujifilm Wako Pure Chemical Corporation, Osaka, Japan).

#### 2.4.2. Gas Chromatography Analyses

The serum sample preparation and the measurement by GC-MS followed the same method as described previously by Trimigno*,* et al. [28] to obtain GC-MS data. Briefly, 100 µL of serum was analysed with a two-step derivatisation (methoximation and silylation) on a GC-MS 7890B/MS5977A (Agilent Technologies, Santa Clara, CA, USA) with a CombiPAL autosampler (CTC-Analytics AG, Zwingen, Switzerland) and a DB-5 ms fused silica capillary column (60 m, 0.25 mm i.d., 0.25 µm film thickness, Agilent Technologies, Basel, Switzerland). Lactose, galactose, galactonate, galactitol, maltose, and 17 amino acids were identified in the dataset, and a relative quantification was performed using an in-house reference compound library, retention indices (RI), and spectral data as described in Munger*,* et al. [29]. Signal drift correction was performed with R (v 4.0.3; R Foundation for Statistical Computing, Vienna, Austria) via the QC-based robust locally estimated scatterplot smoothing signal correction method [30].

Sixty-seven free fatty acids (FFA) were determined by high-resolution lipid analysis using gas chromatography with flame ionization detector (GC-FID) (6890N, Agilent Technologies, Basel, Switzerland; Table S3). Serum samples were prepared for analysis by addition of 30 µL of internal standard (C13, 15 µg/30 µL) to 100 µL of serum, followed by methylation of FFA with MeOH/HCl (25 °C for 45 min). A post-reaction treatment for neutralisation was applied with 350 µL of Na_2_CO_3_ solution (30% in water) and extraction was performed with 300 µL hexane. Serum samples were injected at volumes of 0.5 µL and analyzed analogously to the method published by Collomb, et al. [31]. In total 81 features (67 single FFA and 14 sum parameters) were quantitatively analysed.

### 2.5. Microbiota Analyses

Extraction of faecal DNA from all samples collected for the full study was performed using MagNA Pure automated platform (Roche, Basel, Switzerland) prior to V3V4 16S rRNA library preparation following the Illumina^®^ protocol ‘16S Metagenomic Sequencing Library Preparation’ (Part. #15044223 Rev. B, Illumina^®^, San Diego, CA, USA). Samples were sequenced by Illumina^®^ MiSeq with V3 Reagent kit to generate 2 × 300 bp paired-end reads. Reads were processed as described previously [32], using an in-house Snakemake pipeline (https://github.com/metagenlab/microbiome16S_pipeline accessed on 1 March 2021, release v.0.9.15, [33]). Briefly, reads were trimmed with cutadapt (v 2.10, [34]) and processed by DADA2 (v 1.12.1, [35]) into amplicon sequence variants (ASVs). Taxonomic assignment was completed with the EzBioCloud database (2018.05 release, pre-processed to unite the taxonomic identifier of sequences on the V3V4 fragment, [36]) using the RDP classifier in QIIME (v 1.9.1 [37]). Processed data were imported as a Phyloseq object (v 1.26.1, [38]), where only bacterial ASVs classified at the phylum level were retained and counts were normalised by rarefaction to the minimal number of reads measured for one sample (*n* = 78,000). ASV counts regrouped from species to phyla were considered for statistical analyses. No-template negative controls were included during DNA extraction and library preparation to detect contaminants and yielded a low number of reads (27–346).

Functional metagenomics prediction was performed from ASVs using the PICRUST2 pipeline that after assigning ASVs to reference genomes, extracts functional annotation information from these reference genome (v.2.3.0 b, [39]). Functional information for KEGG [40] pathway-level annotation was extracted from this analysis.

A targeted analysis on 107 species previously identified from fermented food origin by Pasolli, et al. [41] was also conducted. Genomes corresponding to the species identified in the study were downloaded from NCBI Assembly. V3V4 fragments were extracted *in silico* from these assemblies by Simulate_PCR [42] and processed as they would be in our read-processing pipeline to confirm the taxonomic classification of the species concerned.

### 2.6. Statistical Analysis

All data was processed in the R environment (4.0.3, [43]). Baseline differences between the age groups were assessed for all parameters by comparing V1 samples from the YA and OA groups. The effect of the SC phase in the two groups was evaluated by comparison of samples from V3 with respect to V1.

#### 2.6.1. Statistical Analyses on Dietary and Circulating Markers

Non-parametric robust statistical tests were used in this study due to many variables not showing a normal distribution. Differences in age group at baseline were assessed for all dietary and circulating variables by a Wilcoxon signed-rank test (non-paired, *p* < 0.05) (Wilcoxon 1). Exploratory analyses of the global dietary changes from OB to SC phase in the two age groups were performed by principal component analysis (PCA) scaled to unit variance, using FactoMineR (v.2.3) [44] and factoextra (v1.0.7) [45]. To identify significant age, intervention, and interaction effects for the SC phase, non-parametric analysis of longitudinal data (nparLD, v2.1, [46]) was performed for all parameters (using *p* < 0.05 as the Wald Chi-Squared test significance cut-off). For those that showed significant differences in age or diet and/or an interaction effect, a Wilcoxon signed-rank test was used to evaluate variables responding to the intervention in the YA and OA groups separately (paired, *p* < 0.05) (Wilcoxon 2) as well as to identify those that were different by age group in SC phase separately (non-paired, *p* < 0.05) (Wilcoxon 3).

As a significant reduction of body weight and BMI was observed during the SC phase compared to the OB phase, the statistical analyses for the clinical biochemistry were repeated with adjustment for BMI using the residual method [47]. In this method, the residuals of each participant were assessed by a regression model in which measured BMI is the independent variable and the measured marker is the dependent variable. A BMI- adjusted value for the measured marker is derived by addition of the calculated residual to the predicted value for the parameter in the model based on the mean BMI of the population. This allowed us to confirm that the observed changes in clinical biochemistry were not driven only by the BMI change.

The specific impact of the dietary changes on the clinical markers was assessed by correlation analyses performed for parameters that were significantly modulated by the SC phase (nparLD treatment effect *p* < 0.05), with macronutrients included as absolute gram intakes. For each parameter, their relationship with the food groups, food subgroups, and nutrients was assessed separately by Spearman’s correlation using false discovery rate (FDR) criteria for significance (FDR < 0.05) [48] and presented visually using the R package pheatmap (v1.0.12) [49].

#### 2.6.2. Statistical Analyses on Microbiota Data

The effects of dietary intervention and age on microbiota diversity was first evaluated by α-diversity using Shannon [50] and inverse of Simpson indices [51]. As for the dietary and serum analyses, group differences in α-diversity at baseline were assessed by a non- paired Wilcoxon signed-rank test while nparLD models were applied to identify significant age, intervention, and interaction effects of the SC phase (*p* < 0.05). Secondly, the effects of age and the dietary intervention on β-diversity were assessed using Jaccard and Bray distance metrics, performing an analysis of variance using the function *adonis2* (Vegan Package v.2.5.6, [52]). Baseline assessments compared age groups while the effect of the SC phase was assessed by modelling the age and dietary intervention (‘treatment’) effects, with subject identity controlled for using the ‘strata’ option. A *post hoc* pairwise multilevel *adonis* model was used where multiple treatments were compared to identify which conditions were significantly different (*p* < 0.05). All diversity analysis was carried out using rarefied, unfiltered data (*n* = 78,000 reads/sample).

Univariate analysis was completed using microbiota data filtered at the ASV level to remove very low abundance taxa, applying a minimum criteria of presence (>0 counts) in >1% of all samples (3264/5529 ASV, 1271/1532 species, 400/480 genera, 72/105 families, 39/52 orders, 25/31 classes, 14/15 phyla). The package ALDEx2 (v. 1.22.0, [53,54,55]) was used to perform Monte Carlo sampling (*n* = 128) for each sample using Dirchlet distribution and each instance was converted using centred log-ratio (CLR) transformation. This transformed data was used for the subsequent univariate comparisons. Univariate analysis comprised assessment of the group effect at baseline with unpaired Wilcoxon signed-rank test and using nparLD models to identify significant effects of age, intervention, and interactions of the SC phase. Taxa present in <5 samples under investigation were not evaluated. The risk of false positives due to multiple testing was considered by using FDR criteria for significance (FDR < 0.05) [48]. Results were visualised using ggtree (v 2.4.0, [56,57,58]) and an adapted version of the *ggdiffclade* function (MicrobiotaProcess package, v.1.2.0, [59]).

Changes in the predicted functions of bacteria after the SC phase were also assessed for the full cohort using the KEGG annotations obtained by PiCRUST2 analysis. Changes in the relative counts assigned to each KEGG Orthology (KO) function were assessed by using ALDEx2 to transform the data (as described for the univariate analyses) and then comparing the treatment days using a paired t-test. Genes were then ranked by their T-statistic and a geneset enrichment analysis (GSEA) [60] was performed to assess enrichment score (ES) using a weighted Kolmogorov Smirnov test to assess the ranking of the genes in selected reference genesets compared to uniform distribution of the genes within the geneset. Metabolic KEGG pathways were used as the reference genesets (*n* = 109, minimum of 5 genes present/pathway) (https://www.genome.jp/kegg/pathway.html, accessed on 10 November 2020 via Pathview package (v1.30.0) [61]). Differences in the number of genes per geneset were accounted for by the calculation of a ‘normalised’ ES (NES). The NES was compared to random permutations of the ranked gene list (*n* = 10,000 permutations) to assess the significance of the enrichment. Correction for multiple testing was completed by FDR adjustment (FDR < 0.10).

## 3. Results

### 3.1. Dietary Characteristics

During the OB phase, both groups reported a regular dairy product intake in their habitual diet, with median intakes of more than three portions per day (YA: 3.1 (Q1: 2.3, Q3: 4.9) portion/d, OA: 3.8 (Q1: 2.7, Q3: 4.3) portion/d), where one portion is defined as 150 mL milk, 125 g fresh fermented dairy products, and 30 g cheese. There were no significant differences in subgroups of dairy product intake between the YA and OA groups (Table 1). Fermented foods accounted for 25.7% (Q1: 19.1, Q3: 33.2) of total food intake (g/d) in the whole population. Intakes of nine non-dairy food subgroups out of 40 consumed by the subjects were significantly different between the YA group compared to OA group: intakes of bread products, fruits, soups, sugars and honey, vegetable fats and condiments were higher in the OA group compared to the YA group whereas pasta, rice and cereals, red meat, and soft drink intakes were higher in the YA group (Table 1). Total energy and macronutrient intakes were not significantly different between the YA and OA groups during the OB phase (Table 2). Although a lower contribution of protein intake to total energy intake was observed in the OA compared to the YA group, both groups presented a balanced distribution of carbohydrate, protein, and fat intakes during the OB phase based on the national reference intakes in France [62]. Intakes of micronutrients were broadly similar in the YA and OA groups during the OB phase, though significantly higher intakes of vitamin A and folate were observed in the OA compared to the YA group.

**Figure 2:**
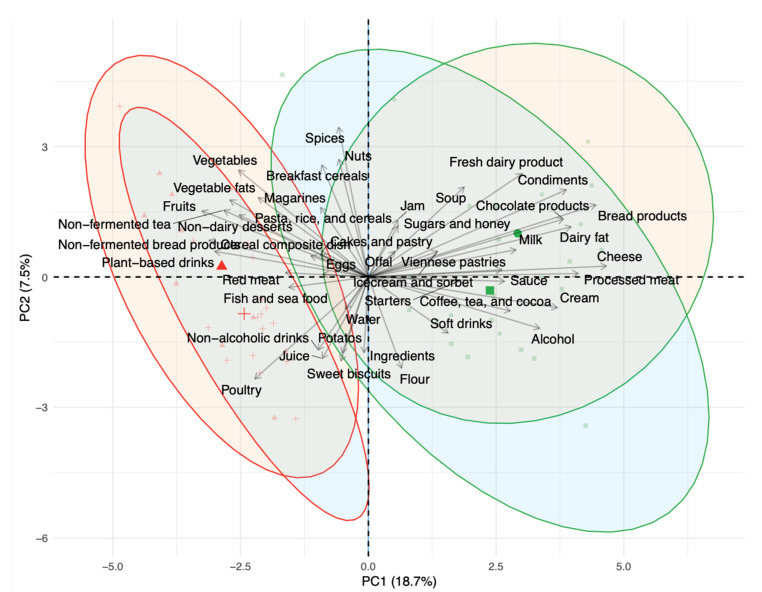
Biplot of principal component analysis (PCA) with a loading plot of daily intake of total food groups for the young adults (YA) and older adults (OA) groups during the observation (OB) and semi-controlled diet (SC) phases. Symbols define the group and period: circles OA, OB phase; squares YA, OB phase; triangles OA, SC phase; crosses YA, SC phase. Ellipse outline represents the period: OB phase in green, SC in red. Ellipse fill represents the group: OA group, orange; YA group, blue. PC, principal component.

**Figure 3:**
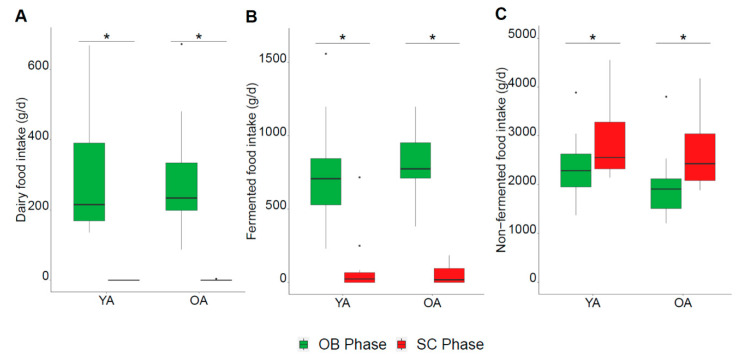
Overview of dietary modifications during the SC phase. (**A**–**C**): daily intake of dairy products, fermented foods, non-fermented foods respectively; OB phase in green, SC phase in red. * Significant difference between the OB and SC phases (diet effect, *p*-value < 0.05) by paired Wilcoxon signed-rank test.

The dietary restrictions resulted in a similar shift in the global dietary pattern of both age groups during the SC phase (Figure 2 and Figure 3; Figure S2) despite a few specific differences in food subgroups (Table 1). As expected, intakes of dairy products and fermented foods were significantly decreased in both the YA and OA groups, confirming their compliance with the dietary restrictions (respectively Figure 3A: diet effect *p* < 0.001, age effect *p* = 0.38, interaction *p* = 0.78; Figure 3B: diet effect *p* < 0.001, age effect *p* = 0.62, interaction *p* = 0.12). In parallel, the participants increased their intakes of non-fermented foods during the SC phase (Figure 3C: diet effect *p* < 0.001, age effect *p* = 0.10, interaction *p* = 0.33) including significant increases in fruits, margarine, non-dairy desserts, non-fermented bread, non-fermented tea, plant-based drinks, meat (poultry and red meat), vegetables, flour, and vegetable fats (Table 1). An interaction between age and diet was observed in nine food subgroups (Table 1), revealing some age-specific adaptations of diet. For example, a greater decrease in bread products intake was found in the OA group, compared to the YA group relating to the significantly higher intakes in the OA group at baseline. This was mirrored in the greater increase in non-fermented bread products during the SC phase for the OA compared to the YA group, though significant increases were confirmed in both groups. Similar age-diet interactions were observed for non-fermented tea which was significantly increased in the OA but not the YA group, while the significant decreases in the fermented coffee and tea group tended to be more marked in the OA group though the age-diet interaction was not significant (*p* = 0.09).

Collectively, these modifications induced a reduction in macronutrients and alcohol intake during the SC phase, resulting in lower total energy intakes in both groups (Table 2). This energy reduction was significantly greater in the YA compared to the OA group: −27.8% (Q1: −36.2%, Q3: −18.5%) reduction in the YA group and −17.9% (Q1: −22.0%, Q3: −11.3%) reduction in the OA group (*p <* 0.05). Dietary fat intake was significantly decreased in both groups though age group influenced the response, with the reduction being greater in the YA group. The decrease in fat intake was largely attributable to the decrease in saturated fatty acid (SFA) intake, resulting in increases in the relative (%) energy intake from monounsaturated fatty acids (MUFA) and polyunsaturated (PUFA) though absolute intakes of these fats were not significantly changed during the SC phase. An interaction between age and diet was observed for total MUFA intake that tended to decrease in the YA while increasing in the OA group leading to a significantly higher levels in OA after the SC phase. Although absolute carbohydrate intake decreased in the YA group and was not significantly changed in the OA group, its relative contribution to total energy intake increased during the SC phase in both groups. On the other hand, absolute protein intake was significantly decreased during the SC phase though its contribution to energy intake was not significantly changed. It is noteworthy that despite the increases in fruit and vegetables intakes, total fibre and sugar intakes were not significantly modified by the SC phase. Modifications in micronutrient intake were observed during the SC phase in both age groups including significantly decreased intakes of calcium, sodium, phosphorus, iron, potassium, magnesium and vitamins B1 and B2, and increased intakes of vitamins C and E. An effect of age on the micronutrient changes was only identified for calcium with greater reductions of intake in the YA than the OA group.

### 3.2. Biochemical Parameters and Serum Metabolites

There was no significant difference in BMI between the two age groups before the intervention despite a significantly higher body weight in the YA group (Table 3). Fasting clinical parameters for the YA and OA groups were also in normal clinical range before the intervention, though the OA group had higher levels of total cholesterol, LDL, and CRP, but lower levels of adiponectin compared to the YA group (*p* < 0.05).

The dietary changes of the SC phase led to small but significant decreases in BMI and body weight that were not age-specific (Table 3). These changes were accompanied by significant diet-related decreases in fasting serum levels of glucose, insulin, total cholesterol, LDL, and HDL. While higher levels of total cholesterol and LDL were observed in the OA group before and after the SC phase, the effect of the diet on the markers were not different between the age groups. Significant diet-age interactions were observed for the relative ratios of total cholesterol/HDL and LDL/HDL but not for total cholesterol/LDL. The different, non-significant effects of the diet for the two groups resulted in significantly lower total cholesterol/HDL and higher LDL/HDL ratios after the SC phase in the OA group compared to the YA group. Neither triglycerides nor NEFA levels were significantly affected by the dietary intervention or showed differences between the age groups. Similarly, no significant effects of age or diet were observed for lactate or urea. The age-related differences observed in the OB phase for CRP and adiponectin were maintained during the SC phase with no significant effects of the diet. All significant effects of treatment were confirmed after adjustment for BMI (data not shown).

Among the biochemical outcomes that showed significant diet effects during the SC phase (*p* value < 0.05), only HDL was significantly associated with changes in specific dietary items. Specifically, HDL was significantly positively associated with dairy fats intake (in particular from butter and other dairy fats groups) (rho = 0.68, *p* = 0.00009, FDR = 0.01) and a non-significant association was observed with cheese (rho = 0.42, *p* = 0.03, FDR = 0.86), while neither fermented fresh dairy products nor milk intake showed any associations (Figure S3). Total dairy intake was not associated with the HDL changes (Figure S4). The reduction in total dairy intake was not associated with the change in total circulating cholesterol or LDL levels (Figure S4) while the reduction in cheese intake showed a non-significant association with total cholesterol (rho = 0.54, *p* = 0.004, FDR = 0.55) (Figure S3). No significant associations with single nutrients were identified (Figure S5).

The targeted analysis of serum metabolites revealed several groups of metabolites that were differently modulated by diet and age (Table 4). Among the selected metabolites associated with carbohydrate metabolism, two derivatives of lactose metabolism, galactitol and galactonate, were significantly higher in the OA compared to the YA though lactose levels were not different between the groups and the related intermediate galactose levels were below the limit of detection in all samples. Conversely only lactose was significantly decreased during the SC phase while galactitol and galactonate levels were not significantly modulated by the dietary changes.

No significant differences in fasting amino acid levels were observed between the groups. However, among the 17 identified amino acids, three amino acids (tyrosine, leucine, and valine) were significantly decreased by the dietary intervention, the changes in leucine and valine driving a significant decrease in total branched-chain amino acids (BCAA). The changes in these few individual amino acid levels, did not change the total amino acid levels.

In contrast to the amino acid profiles, FFA levels were globally different between the OA and YA groups during the OB phase, including total FFA, SFA, PUFA, as well as branched-chain fatty acids (BCFA), and the odd-chain fatty acids (OCFA) pentadecanoic and heptadecanoic acids (Table 4 and Table S3). Short-chain fatty acids (SCFA) were only present at very low levels and did not show significant differences by age, in contrast to medium-chain fatty acids (MCFA) and long-chain fatty acids (LCFA) which were both higher in the OA compared to the YA group. Specific fatty acids were found to be especially discriminant of age (Table S3). For example, individual SFA that showed significant differences between the age groups were MCFA with 15 to 18 carbons (C15:0–C18:0) while total unsaturated fatty acids and MUFA were not significantly different between the OA and YA groups, total PUFA levels were higher in the OA group in the OB phase, with significant differences observed for the longer chain PUFAs such as EPA, DPA, and DHA. 

Interestingly, individual BCFA that were significantly different by age included those based on a 15 and 17 carbon fatty acid but only *iso*- BCFA and not *anteiso*-BCFA, i.e., 13-methyltetradecanoic acid (*iso*-C15:0) and 15-methylhexadecanoic acid (*iso*-C17:0). Despite the changes in the levels of individual FFA, their total quantity was not significantly modified during the SC phase in either group. Even though total SFA were not significantly reduced by the dietary intervention (Table 4), circulating levels of a number of SFA, specifically pentadecanoic acid (C15:0), octadecanoic acid (C18:0), and docosanoic acid (C22:0), were significantly reduced (Table S3). The SC phase resulted in significant increases in total circulating levels of USFA, including MUFA, while other fatty acids, in particular BCFA and CLA, were significantly reduced by the SC phase (Table 4). Of the PUFA, only the omega 3 sub-group contributed to the increase in unsaturated fatty acids (Table 4). Conversely, four individual PUFA and one isoform of CLA were significantly decreased by the SC phase, independently of age group (Table S3). Of note, changes in the global relative composition of circulating fatty acids reflected the changes in dietary lipid intake during the SC phase (Table 2), with a significantly decreased proportion of SFA and an increased proportion of MUFA in serum, while the proportion of PUFA did not change (Table 4).

### 3.3. Microbiome Analyses

The gut microbiota analyses of faecal samples from the OB phase confirmed the high inter-individual variability of the gut microbiota composition [63]. No significant differences were found in the global gut microbiota composition at baseline between the YA and OA groups either by evaluation of α-diversity (Observed richness YA: 347 (Q1: 254, Q3: 387) vs. OA: 370 (Q1: 266, Q3: 420), *p* = 0.10; Shannon index YA: 4.2 (Q1: 4.1, Q3: 4.3) vs. OA: 4.3 (Q1: 3.8, Q3: 4.6), *p =* 0.58) or β-diversity (Jaccard, *p* = 0.18, Bray, *p =* 0.35). In addition, no differences between the YA and OA groups were found for the relative abundance of bacteria at any taxonomic level (FDR > 0.05).

The α-diversity of the samples, as assessed by Observed richness and Shannon index, was not changed significantly after the SC phase compared to the OB phase. While no significant differences were observed for β-diversity, as measured by Jaccard distances (based on presence/absence of ASVs), the combined community structure characterised by Bray distances (based on relative abundance of ASVs) was modified significantly in response to the dietary intervention of the SC phase (*p =* 0.001) with an age effect (*p =* 0.001). Considered separately, the β-diversity for both the YA and OA groups was significantly changed by the dietary intervention (*p <* 0.05) as well. The importance of the change in microbiota composition, as measured by the median Bray distance between V1 and V3 samples, was not significantly different between YA and OA subjects (YA: 0.38 (Q1: 0.30, Q3: 0.44) vs. OA: 0.42 (Q1: 0.41, Q3: 0.45), *p >* 0.05).

**Figure 4:**
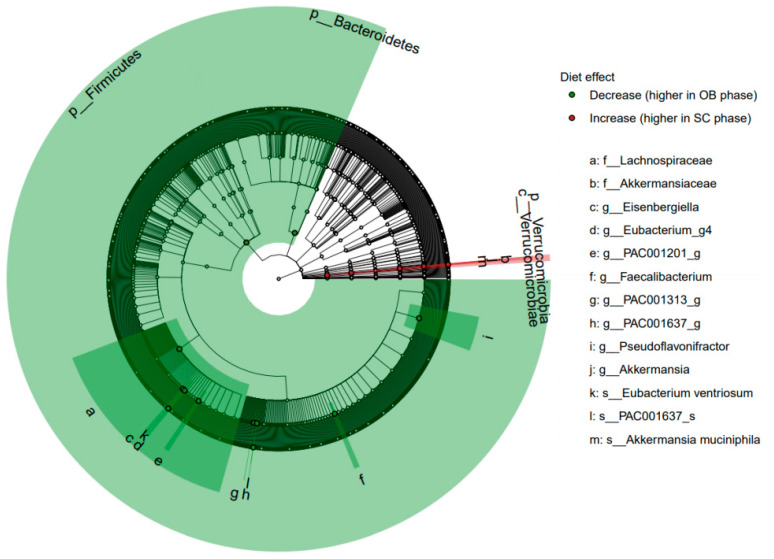
Cladogram for all subjects and 17 taxa showing significant microbiota changes between the observation (OB) and semi-controlled diet (SC) phases (relative increases in the SC phase in red, decreases in green). Bacteria that show a significant change (FDR < 0.05) are highlighted with a node circle and labelled with text and clade. Taxonomic level indicated by letter preceding the bacterial descriptor from genus, ‘s_’ to phyla, ‘p_’.

**Figure 5:**
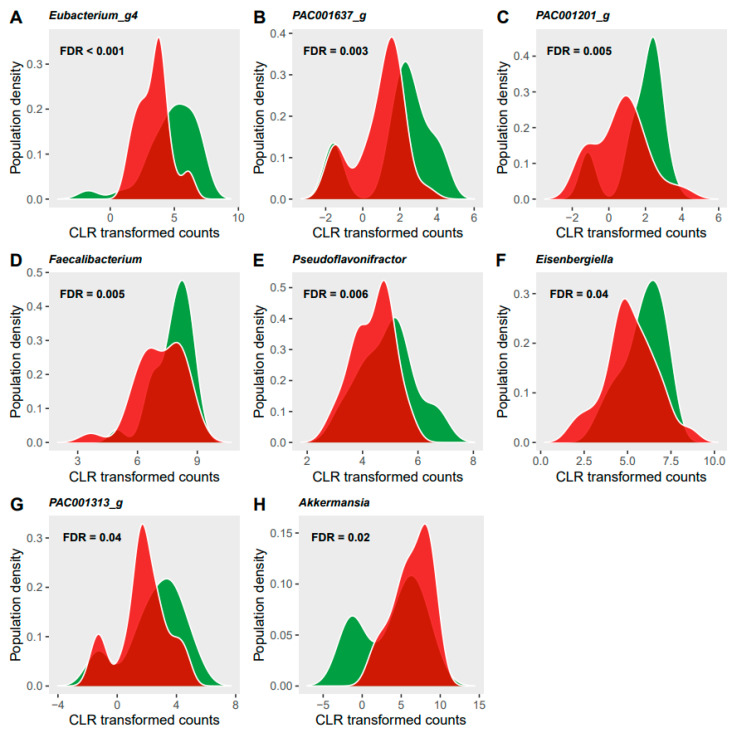
Significant changes in the relative abundance of eight genera (**A**–**H**) in response to the semi-controlled diet. *X* axis is the relative abundance of the bacterium given in centred log-ratio (CLR) transformed counts and *Y* axis is the prevalence of participants. The sample distribution of the observation phase is illustrated in green and of the semi-controlled diet phase in red.

The relative abundance of seventeen bacterial taxa (three species, eight genera, two families, one class, three phyla) was significantly modified by the dietary restrictions (FDR < 0.05, Figure 4). These changes were not significantly different between age groups with no significant interactions found between age and diet. Two species of the Clostridiales order were significantly decreased during the SC phase in response to the restrictive diet: *Eubacterium ventriosum* (FDR < 0.001) and PAC001637_s of the family Ruminococcaceae (FDR = 0.006). These effects were also observed for the respective genera of these species as well as five other genera in the Clostriales order (Figure 5, panels A-G). These modifications collectively contributed to a decreased relative abundance of the *Lachnospiraceae* family (FDR = 0.02), Clostridia class (FDR = 0.06) as well as the Firmicutes phylum (FDR = 0.008) in response to the restricted diet. The Bacteroidetes phylum also decreased significantly during the SC phase (FDR = 0.03). Only one species, *Akkermansia muciniphila* showed a significant increase during the SC phase compared to the OB phase (FDR = 0.04), with its relative abundance doubling in the YA group (V1: 6.7 (Q1: −1.3, Q3: 10.5) vs. V3: 12.5 (Q1: 7.2, Q3: 13.5)), and increasing by 30% in the OA group (V1: 6.8 (Q1: −1.2, Q3: 12.1) vs. V3: 9.2 (Q1: 5.1, Q3: 12.5)). The increase in *Akkermansia muciniphila* was responsible for significant changes at all taxonomic levels of the species from genus (FDR = 0.03, Figure 5F) to phylum (FDR = 0.02) levels with the exception of order level.

Fifteen of the 107 faecal bacterial species previously identified as of food origin [41] were present in the filtered dataset (>1% of the samples) with six meeting the criteria for univariate analysis (*Bifidobacterium longum*, *Enterobacteriaceae* sp., *Escherichia* sp., *Enterobacter cloacae, Lactococcus lactis, Streptococcus thermophilus*). Targeted analyses of these species revealed that *Streptococcus thermophilus* decreased during the SC phase (*p* = 0.007).

Based on the predicted gene presence in the bacteria, modifications of functional pathways in the gut microbiota were observed. The SC diet led to a change in microbiota functionality characterised by an enrichment of bacteria predicted to carry genes involved in oxidative phosphorylation (NES = 1.47, *p =* 0.001, FDR = 0.09).

## 4. Discussion

Excluding dairy and fermented foods from the diet of young and older adult men for 19 days led to substantial changes in dietary pattern and nutrient intake. These changes resulted in modifications in clinical metabolic parameters, circulating levels of nutrient-related serum metabolites, and gut microbiota composition of the participants. Decreased circulating markers of energy metabolism, particularly lipid metabolism, accompanied the reduction in macronutrient intake, while the reduction in dairy foods was reflected in the decreased levels of several candidate biomarkers of dairy intake. Some lipid species, such as unsaturated fatty acids were specifically increased following the dietary restrictions, reflecting the compensatory changes in dietary pattern. The composition of the gut microbiota was also modified by the diet, as assessed by the Bray β-diversity distance between samples collected before and after the intervention. This overall change in composition was associated with a significant increase in relative abundance of *Akkermansia muciniphila* and a decreased relative abundance of bacteria of the Clostridiales order and *Streptococcus thermophilus*. Despite the fact that the OA group was clearly distinguished from the YA group by their higher levels of circulating lipids and cholesterol profiles, the diet-driven changes in clinical parameters, serum metabolites, and gut microbiota composition were generally similar for both groups, suggesting that the effect of the dietary modification was more pronounced than the impact of age and, consequently, that the candidate diet-sensitive markers identified in this study could be representative of both age groups.

To our knowledge, this is the first study in which two different age groups of ‘free-living’ individuals were asked to shift from their normal diet (rich in dairy products and fermented foods) to a diet without dairy products and restricted in major dietary sources of probiotics (fermented foods). As the dietary modifications influenced the intake of various food groups to compensate the restricted dairy and fermented foods, the interpretation of the results considers the global dietary pattern shift rather than only that of one specific type of food intake.

### 4.1. Serum Metabolites Modification by Exclusion of Dairy and Limitation of Fermented Foods

In this study, the exclusion of dairy foods from the diet and the reduced intake of fermented foods resulted in significant modifications of food and nutrient intakes. Collectively, these dietary changes led to decreased energy intake due to decreased intake of all three macronutrients that were reflected in the decreased circulating levels of metabolites derived from these nutrients. Remarkably, many of these metabolites have been associated with the composition and intake of dairy products, including pentadecanoic acid [28,64], BCFA [65], lactose [28,29], and BCAA [66,67].

The significant decrease in total fat intake, in particular SFA, was a major factor contributing to the reduction in energy intake in the participants. As dairy products are an important source of dietary fat, accounting for 17% of total fat intake in France [68], the exclusion of dairy products could partly explain the significant decrease in fat intake observed in our study. However, the decreased intake of fermented foods, such as processed meats, could also have contributed to the decreased fat intake. Consistent reductions were observed for various circulating fatty acids that are enriched in dairy products, including BCFA, CLA, as well as certain SFA. In particular, pentadecanoic acid (C15:0) and CLA, both known candidate biomarkers of dairy fat intake [69,70], showed significant decreases after the SC phase. However, the specificity of these markers for the intake of dairy products needs to be more precisely evaluated, in particular in light of their presence in meat products [69].

It was remarkable that total cholesterol, HDL and LDL were also significantly reduced by the diet in the context of the circulating FFA that were reduced. These cholesterol changes were associated with certain dairy foods, in particular for HDL and dairy fat, but not with other dairy foods or total dairy products. Although dairy products [71], including dairy fat [72], have been associated with improved profiles of circulatory cholesterol, the magnitude of these effects remains moderate and their impact on health should not be overestimated, in particular in light of the revaluation of the impact of fats and dairy fats on cardiovascular disease [73]. In addition, serum HDL levels of all participants remained in the healthy physiological range.

Not all fatty acids were significantly reduced by the diet and the differential changes in specific circulating lipids offered insights into the distinct dietary adaptations that also comprised increases in some sources of non-dairy fats. Notably, increases in circulating unsaturated fatty acids, in particular MUFAs, seemed to correspond to the increased use of non-dairy fats.

The dietary changes in protein were more complex to capture by circulating amino acids, which showed fewer specific diet-related changes than the circulating lipids. Significant decreases of BCAA levels were observed as these amino acids are particularly enriched in dairy proteins that were eliminated from the diet by the intervention. However, these amino acids are also enriched in proteins from other food groups like meats, which were also modified by the dietary intervention. Moreover, although BCAAs such as valine increase postprandially after intake of dairy products [67], a one-month intervention in healthy subjects changing dairy protein intake did not significantly modify the circulatory levels of BCAA [74]. On the other hand, the reduction on BCAA levels could also relate to the improved glucose homeostasis observed after the SC phase [75]. Finally, endogenous amino acids can also participate in defining the circulating pool of amino acids and thus limit the use of these metabolites as dietary biomarkers.

The intervention imposed significant changes in the major sources of dietary carbohydrate, which shifted from dairy products and fermented bread to fruits and non-fermented bread. A specific reduction in the dairy-rich sugar lactose was confirmed by the significant decrease of the disaccharide in serum following the restricted diet. Lactose has previously been proposed as a candidate biomarker of dairy intake, evidently for products in which the disaccharide has not been removed or reduced technologically [29]. However, given that its presence in serum is low and influenced by lactose tolerance status [76], its utility remains to be defined. Apart from molecules associated with lactose, no specific biomarkers were available to evaluate the overall effect of the carbohydrate reduction but it was remarkable that both circulating glucose and insulin were significantly reduced after the SC phase. These effects were maintained after adjustment for BMI change suggesting that dietary change was the cause of their modulation. The amounts and composition of many foods and nutrients in the diet can influence glucose homeostasis, as reported for fruits, vegetables, fibre, protein, fats, and minerals [77]. However, there is now considerable evidence to suggest that dietary effects on glycemia are highly individualised, particularly during the postprandial phase after eating, challenging the notion of single glucose lowering diet [78]. It was thus not surprising that, in the context of both the specific dietary changes required from the study participants and the more variable compensatory changes that the participants implemented for the dietary restrictions, no single food groups, subgroups or nutrients explained the decreased glucose and insulin levels. In addition, other parameters notably the gut microbiota and its metabolites could have modulated the individual response to the dietary change.

### 4.2. Gut Microbiota Modifications and Its Potential Associations with Diet and Circulating Metabolites

In response to the diet imposed during the SC phase, we observed significant changes in the microbiota composition. The withdrawal of dairy and fermented food intake induced a significant modification in the relative similarity of gut microbiota communities (β-diversity) in both age groups, but the diversity of individual samples (α-diversity) was not significantly changed. Consistent increases of the relative abundance of *A. muciniphila*, a mucin degrading bacterium of the phylum Verrucomicrobia were observed in this study, in agreement with previous studies in which fruit and vegetable intake has been increased [3,79]. *A. muciniphila* has been widely investigated for probiotic properties including its impact on circulating glucose, insulin, and triglycerides levels [80], the regulation of cytokine expression, and the lowering of intestinal permeability by thickening the mucosa layer [81]. The decreased levels of glucose and insulin observed during the SC phase could thus, in addition to the effect of the metabolites and dietary changes discussed already, be partially attributed to the increased abundance of this species.

The abundance of bacteria of the Clostridiales order was decreased during the SC phase. Many of these bacteria have known metabolic capacity to produce butyrate, including bacteria of the *Ruminococcaceae* and *Lachnospiraceae* families [82,83]. Several dietary changes could have played a role in these decreases. Notably the intake of carbohydrates is a major driver of the levels of butyrate-producing bacteria [84] and could thus have contributed to these changes. Recently, supplementing rats with calcium and magnesium was shown to increase the intestinal abundance of *Ruminococcaceae* and the concentration of butyrate [85], suggesting that the decrease intake of these minerals observed in our study might have also contributed to the decrease in bacteria of the Clostridiales order. However, none of the SCFA were significantly changed in serum during the SC phase. How these factors finally balance each other to determine the net amounts of butyrate and other SCFA produced by the gut microbiota could eventually be clarified by a determination of these metabolites in faecal samples.

### 4.3. Different Modifications between Young and Older Adults in Response to the Restriction on Dairy and Fermented Food Intake

The participants in this study were selected to represent two age groups, healthy young and older adult men. Higher levels of total cholesterol, LDL, and CRP were found in the OA group compared to the YA group in the current study in agreement with published studies as reviewed by Costantino, et al. [86]. The OA group also showed higher levels of some diet-related serum metabolites such as higher circulating levels of galactitol, galactonate and FFA. Galactitol and galactonate are produced from galactose by aldose reductase but cannot be further metabolised and are subsequently excreted in urine [87]. Ageing is accompanied by physiological changes in renal function including a decrease in glomerular filtration rate and renal plasma flow [88]. Decreasing excretion of galactitol and galactonate with age has been observed in previous studies [89,90] possibly explaining the higher levels of these markers in the OA group. Higher levels of FFA in blood were also observed in the OA group, a well-known characteristic of ageing that is attributable to an increased release of FFA from adipocytes and a decreased cellular capacity to oxidise them [91]. Interestingly, some SFA associated with dairy (for example pentadecanoic acid and BCFA) were significantly higher in the OA than the YA group during the OB phase that seemed to correspond to the non-significant trend for the OA group to consume higher amounts of dairy fats than the YA group. However, this did not extend to a significant age mediated response to the SC phase.

Unlike previous studies [63,92], the gut microbiota profile did not show significant differences between the YA and OA groups at baseline. The nutrient profile of the two age groups also did not reveal major differences in dietary intake. In addition, the population from which the OA group was recruited was healthy and the participants were selected by strict health criteria; this removed some of the typical ageing characteristics, such as hyperglycaemia and intake of medication, that are associated with differences in gut microbiota composition in older populations [93]. Diet and health status therefore likely explain the overall similarity in gut microbiota composition between the two age groups at baseline.

### 4.4. Limitations of the Study

While this study offers some insights into the effects of a major dietary change on multiple biological outcomes in two distinct age groups, it has several limitations. Firstly, the intervention made during the SC phase was complex from a nutritional point of view, including not only restrictions in two overlapping groups of foods, namely dairy products and fermented foods, but also, as a consequence of these restrictions, compensatory increases in other food groups such as fruits and vegetables. In addition, the compensation of energy intake and intake of important dairy micronutrients, such as calcium, phosphorus, and vitamin B2, was not fully achieved during the intervention. The results can thus only be interpreted in the context of a global dietary modification rather than of changes in specific food groups. Secondly, it was difficult to interpret the consequence of changes in microbiota composition on microbiota functionality. Indeed, 16S rRNA amplicon-based metagenomics does not allow to directly infer bacterial metabolism but requires the prediction of an expected functional profile based on available bacterial genome sequences from similar species. Hence, bacterial functions remain hypothetical and predictions should be confirmed by alternative methods such as shot-gun metagenomics or functional tests. Finally, the combined withdrawal of dairy products and reduction of fermented foods from the diet is an artificial intervention that does not reflect any normal dietary pattern. However, from a nutritional point of view, the design of the OB and SC phases offered interesting insights into the impact of a short-term intervention with major dietary changes on human metabolism and microbiota. Nonetheless, the results of our study should be interpreted with caution, notably when extrapolating them to the potential long-term effects of such dietary restrictions.

## 5. Conclusions

This study demonstrates the exclusion of dairy and limited intake of fermented foods followed over 19 days induced metabolic changes in both young and older adult healthy men as well as altering the composition and diversity of their gut microbiota. Candidate biomarkers of dairy intake, such as lactose and pentadecanoic acid (C15:0), decreased with the exclusion of dairy foods, supporting their potential use as biomarkers of dairy intake. Fasting levels of cholesterol (total, HDL, LDL), glucose, and insulin also decreased as a result of complex interactions between the intended and compensatory dietary changes. These dietary changes were accompanied by specific modifications in the composition of the gut microbiota, including increased relative abundance of *A. muciniphila*, and decreased relative abundance of bacteria of the Clostridiales order. Each of these changes in the gut microbiota might have also contributed to the effects of the dietary restriction on metabolic parameters. Also, higher baseline levels of free fatty acids (FFA) associated with dairy products were measured in the OA group, which nonetheless decreased in a similar manner in both groups during the intervention. In conclusion, restricting the intake of dairy and fermented foods in men modified their gut microbiota and serum metabolites and the impact of these restrictions on these outcomes was more marked than the effect of age.

## Figures and Tables

**Table 1:** Food subgroup intakes (median (IQR); g/d) during observation and semi-controlled phase in young and older men (foods consumed by at least 25% of subjects).

	YA		OA		*p*-Value (Wilcoxon 1)	*p*-Value (Wald Test) ^a^		
	OB Phase	SC Phase	OB Phase	SC Phase	Baseline OA vs. YA	Age Effect	Diet Effect	Interaction
**Dairy food subgroups**								
Cheese	42.7 (34.0, 67.3)	0.0 (0.0, 0.0) *	51.8 (44.7, 73.0)	0.0 (0.0, 0.0) *	0.220	0.195	**<0.001**	0.195
Cream	6.7 (0.5, 17.2)	0.0 (0.0, 0.0) *	11.8 (4.7, 17.8)	0.0 (0.0, 0.0) *	0.394	0.231	**<0.001**	0.231
Dairy fats	5.0 (1.2, 7.9)	0.0 (0.0, 0.0) *	8.1 (2.6, 18.2)	0.0 (0.0, 0.0) *	0.126	0.097	**<0.001**	**0.041**
Fresh fermented dairy products	102.2 (60.1, 161.5)	0.0 (0.0, 0.0) *	111.1 (63.8, 133.3)	0.0 (0.0, 0.0) *	1.000	0.785	**<0.001**	0.785
Milk	78.7 (4.3, 122.1)	0.0 (0.0, 0.0) *	88.9 (16.1, 127.9)	0.0 (0.0, 0.0) *	0.827	0.682	**<0.001**	0.868
**Non-dairy fermented foods subgroups**								
Alcohol	185.2 (39.9, 259.7)	0.0 (0.0, 0.0) *	147.8 (61.1, 339.4)	0.0 (0.0, 0.0) *	0.903	0.989	**<0.001**	0.900
Bread products	87.8 (55.4, 124.8)	0.0 (0.0, 0.0) *	167.4 (129.4, 197.0) #	0.0 (0.0, 3.3) *	**0.002**	**0.001**	**<0.001**	**0.048**
Cake and pastries	0.0 (0.0, 0.0)	0.0 (0.0, 0.0) *	0.0 (0.0, 5.6)	0.0 (0.0, 17.7) #	0.777	**0.027**	0.887	**0.022**
Chocolate products	2.2 (0.3, 7.4)	0.0 (0.0, 0.0) *	6.1 (4.8, 10.9)	0.0 (0.0, 0.0) *	0.125	0.092	**<0.001**	0.092
Coffee, tea, cocoa	136.0 (37.2, 244.3)	0.0 (0.0, 50.0) *	255.6 (91.2, 420.4)	0.0 (0.0, 0.0) *	0.234	0.642	**<0.001**	0.085
Processed meat	54.8 (44.0, 64.6)	0.0 (0.0, 12.0) *	38.2 (10.2, 58.8)	0.0 (0.0, 6.0) *	0.202	0.257	**<0.001**	0.491
Viennese pastries	4.3 (0.0, 24.6)	0.0 (0.0, 0.0) *	6.7 (0.0, 12.8)	0.0 (0.0, 0.0) *	0.860	NA	**<0.001**	NA
**Non-dairy and non-fermented foods subgroups**								
Breakfast cereals	0.0 (0.0, 21.6)	7.9 (0.0, 17.8)	0.0 (0.0, 0.0)	0.0 (0.0, 12.3)	0.218	0.374	0.066	0.354
Eggs	25.3 (14.8, 55.1)	25.0 (11.2, 42.1)	24.6 (17.7, 34.4)	41.7 (25.0, 50.0)	0.905	0.444	0.159	**0.008**
Fish and seafood	28.1 (16.7, 61.5)	51.4 (14.2, 78.3)	45.4 (26.1, 64.6)	75.2 (29.2, 83.8)	0.234	0.330	0.249	0.959
Fruits	93.9 (50.6, 201.5)	359.4 (249.2, 563.8) *	230.1 (186.8, 293.7) #	295.7 (260.7, 339.7) *	**0.025**	0.382	**<0.001**	**0.021**
Ice cream and sorbet	0.0 (0.0, 16.9)	0.0 (0.0, 0.0)	0.0 (0.0, 0.0)	0.0 (0.0, 0.0)	0.352	0.970	**0.022**	0.117
Ingredients	0.0 (0.0, 0.0)	0.0 (0.0, 0.0)	0.0 (0.0, 0.1)	0.0 (0.0, 0.0)	0.529	0.429	0.683	0.881
Jam	0.0 (0.0, 0.0)	0.0 (0.0, 0.0)	0.0 (0.0, 5.7)	0.0 (0.0, 0.0)	0.067	0.074	0.202	0.278
Juice	47.3 (9.4, 62.8)	44.1 (4.6, 118.7)	14.1 (3.1, 39.8)	59.8 (11.3, 103.8) *	0.307	0.744	0.164	0.269
Margarines	0.3 (0, 0.6)	2.3 (0.0, 4.8) *	0.0 (0.0, 3.6)	6.7 (2.7, 7.7)	0.979	0.286	**0.001**	0.272
Non-alcoholic drinks	0.0 (0.0, 0.0)	0.0 (0.0, 0.0)	0.0 (0.0, 0.0)	0.0 (0.0, 26.7)	0.591	0.430	0.243	0.772
Non-dairy desserts	0.0 (0.0, 0.0)	0.0 (0.0, 91.7)	0.0 (0.0, 0.0)	0.0 (0.0, 166.7)	0.563	0.766	**0.019**	0.813
Non-fermented bread	0.0 (0.0, 0.0)	30.5 (11.6, 53.0) *	0.0 (0.0, 0.0)	54.0 (40.8, 133.0) *#	0.969	**0.045**	**<0.001**	**0.019**
Non-fermented tea	0.0 (0.0, 54.2)	54.2 (0.0, 213.5)	0.0 (0.0, 0.0)	260.0 (100.0, 300.0) *	0.089	0.826	**<0.001**	**0.004**
Nuts	2.4 (0.0, 8.5)	0.0 (0.0, 3.5)	3.3 (0.0, 9.1)	9.2 (0.0, 18.8)	0.654	0.262	0.881	0.290
Pasta, rice, and cereals	126.9 (85.1, 136.1)	137.4 (88.5, 184.1)	85.4 (47.8, 102.6) #	82.5 (42.5, 106.0) #	**0.012**	**0.002**	0.741	0.721
Plant-based drinks	0.0 (0.0, 0.0)	53.1 (0.0, 102.5) *	0.0 (0.0, 0.0)	67.0 (6.7, 96.0) *	0.625	0.919	**<0.001**	0.549
Potatoes	66.4 (44.8, 107.1)	60.8 (34.4, 133.0)	52.3 (41.3, 72.6)	62.0 (31.8, 113.3)	0.423	0.649	0.827	0.659
Poultry	25.3 (15.7, 42.0)	80.0 (46.0, 96.7) *	11.1 (0.0, 26.8)	27.5 (20.3, 48.7) #	0.163	**0.002**	**0.001**	0.402
Red meat	48.2 (34.6, 63.9)	57.7 (46.8, 66.7)	30.3 (23.4, 38.0) #	69.7 (18.5, 96.7) *	**0.044**	0.325	**0.009**	0.238
Soft drinks	97.2 (37.9, 135.4)	0.0 (0.0, 0.0) *	0.0 (0.0, 0.0) #	0.0 (0.0, 0.0)	**0.001**	**0.007**	**0.003**	**0.001**
Soup	0.0 (0.0, 0.2)	0.0 (0.0, 0.0)	11.2 (0.0, 75.6) #	0.0 (0.0, 1.5)	**0.031**	**0.012**	**0.010**	0.505
Spices	0.6 (0.0, 1.2)	0.0 (0.0, 0.7)	0.6 (0.0, 1.0)	0.0 (0.0, 0.8)	0.633	0.846	0.145	0.580
Sugars and honey	5.8 (1.8, 8.2)	1.3 (0.0, 8.1)	13.2 (7.9, 20.3) #	13.7 (6.0, 22.0) #	**0.033**	**0.002**	0.578	0.497
Vegetable fats	8.8 (6.3, 14.4)	20.0 (11.9, 27.7) *	20.3 (10.9, 23.0) #	27.5 (22.3, 34.2) *	**0.026**	**0.033**	**<0.001**	0.592
Vegetables	158.8 (130.1, 178.9)	203.8 (157.4, 226.7) *	210.9 (168.2, 250.3)	276.7 (218.8, 347.5) *	0.068	**0.048**	**0.001**	0.912
Water	1183.4 (766.0, 1812.4)	1216.3 (771.7, 1799.3)	832.9 (765.6, 1066.7)	864.7 (688.7, 1513.5)	0.128	0.140	0.453	0.927
**Non-dairy, fermented/non-fermented foods subgroups**								
Cereal composite dish	0.0 (0.0, 6.4)	0.0 (0.0, 0.0)	0.0 (0.0, 32.0)	0.0 (0.0, 20.2)	0.300	0.181	0.878	0.971
Condiments	3.6 (0.3, 10.3)	0.0 (0.0, 0.0) *	11.1 (8.4, 14.1) #	0.0 (0.0, 0.0) *	**0.027**	0.120	**<0.001**	**0.010**
Offal	0.0 (0.0, 0.0)	0.0 (0.0, 0.0)	0.0 (0.0, 10.0)	0.0 (0.0, 14.3)	0.098	**0.006**	0.675	0.970
Flour	4.7 (0.4, 10.2)	0.0 (0.0, 5.0)	6.4 (1.8, 12.0)	2.3 (0.0, 10.5)	0.465	0.209	**0.045**	0.618
Sauces	10.8 (2.9, 19.1)	0.0 (0.0, 0.0) *	3.3 (2.2, 6.7)	0.0 (0.0, 1.7) #	0.103	0.557	**<0.001**	0.050
Starters	0.0 (0.0, 0.0)	0.0 (0.0, 0.0)	0.0 (0.0, 3.9)	0.0 (0.0, 0.0)	0.176	0.261	**0.016**	0.164
Sweet biscuits	1.3 (0.0, 15.6)	0.0 (0.0, 7.9)	0.0 (0.0, 3.3)	0.0 (0.0, 18.0)	0.379	0.932	0.799	0.274

Differences in age group at baseline were assessed by a non-paired Wilcoxon signed-rank test (*p*-value < 0.05) (Wilcoxon 1); * Significant difference between periods (diet effect, *p*-value < 0.05) by paired Wilcoxon signed-rank test (Wilcoxon 2); # Significant difference between groups (age effect, *p*-value < 0.05) by non-paired Wilcoxon signed-rank test (Wilcoxon 3); ^a^ *p*-value calculated by Wald Chi-Squared test, statistical significance is indicated in bold when *p*-value < 0.05. NA: model was unable to provide an estimate based on the data available. IQR: interquartile range; OA: older adult men; OB: observation; SC: semi-controlled diet; YA: younger adult men.

**Table 2:** Energy, macro- and micronutrients intake (median (IQR)) during observation and semi-controlled diet periods in young and older men.

	YA		OA		*p*-Value (Wilcoxon 1)	*p-*Value (Wald Test) ^a^		
	OB Phase	SC Phase	OB Phase	SC Phase	Baseline OA vs. YA	Age Effect	Diet Effect	Interaction
**Energy and macronutrients**								
Energy, kcal/d	2321 (2021, 2465)	1693 (1319, 1889) *	2433 (2227, 2588)	2000 (1868, 2564) *#	0.583	0.101	**<0.001**	**0.031**
Energy, kJ/d	9710 (8454, 10,314)	7081 (5519, 7901) *	10,178 (9316, 10,827)	8367 (7815, 10,726) *#	0.583	0.101	**<0.001**	**0.031**
Total Fat, g/d	86.1 (74.4, 99.8)	51.2 (36.5, 70.9) *	92.2 (84.7, 98.9)	73.4 (69.8, 95.6) *#	0.616	0.112	**<0.001**	**0.018**
Fat, %E	34.5 (32.9, 37.0)	29.9 (24.5, 33.3) *	35.1 (32.3, 36.1)	32.2 (30.3, 34.6) *	0.867	0.574	**<0.001**	0.342
SFA, g/d	35.3 (29.6, 40.0)	12.6 (8.0, 14.5) *	35.6 (27.3, 45.9)	14.0 (11.5, 18.3) *	0.650	0.173	**<0.001**	0.211
SFA, % of total fat	40.4 (35.2, 41.5)	22.4 (20.8, 24.6) *	41.0 (35.2, 43.9)	19.6 (18.4, 21.9) *	0.830	0.529	**<0.001**	0.192
MUFA, g/d	27.3 (24.5, 32.2)	24.0 (14.4, 32.4)	31.4 (26.3, 36.9)	37.5 (30.0, 42.8) #	0.350	**0.049**	0.723	**0.018**
MUFA, % of total fat	32.0 (30.9, 34.3)	45.1 (39.7, 46.0) *	34.1 (32.4, 37.8)	46.0 (42.7, 50.0) *	0.076	**0.044**	**<0.001**	0.871
PUFA, g/d	10.7 (7.8, 12.2)	9.4 (6.0, 11.5)	12.3 (9.6, 14.1)	11.6 (8.9, 14.7)	0.280	0.187	0.435	0.854
PUFA, % of total fat	10.9 (9.3, 15.1)	17.7 (15.3, 22.1) *	13.5 (11.3, 14.4)	15.7 (13.2, 19.4) *	0.185	0.869	**<0.001**	0.085
Carbohydrate, g/d	214.3 (195.2, 270.0)	184.4 (163.3, 253.1) *	240.9 (230.5, 256.8)	252.5 (199.6, 287.4) #	0.302	0.124	**0.041**	**0.019**
Carbohydrate, %E	41.8 (35.2, 43.9)	48.3 (41.9, 52.0) *	42.8 (39.5, 44.0)	50.1 (47.2, 51.5) *	0.830	0.590	**<0.001**	0.761
Protein, g/d	100.1 (83.7, 115.2)	76.6 (62.2, 99.2) *	94.5 (75.7, 105.0)	85.9 (64.2, 97.1) *	0.259	0.587	**<0.001**	0.313
Protein, %E	17.4 (16.1, 19.3)	20.8 (18.2, 22.5) *	15.4 (14.1, 16.3) #	15.9 (13.8, 16.2) #	**0.033**	**0.002**	0.140	0.236
Starch, g/d	100.0 (80.9, 110.0)	71.8 (54.0, 110.0) *	102.8 (85.5, 127.6)	78.2 (60.3, 114.7)	0.430	0.607	**0.002**	0.745
Sugars, g/d	89.9 (76.7, 101.5)	89.4 (61.9, 101.1)	93.5 (83.5, 100.2)	98.9 (92.7, 116.8)	0.458	0.179	0.231	0.089
Alcohol, g/d	10.2 (2.1, 18.6)	0.0 (0.0, 0.0) *	13.3 (4.8, 32.3)	0.0 (0.0, 0.0) *	0.905	0.878	**<0.001**	0.975
Alcohol, %E	3.3 (0.6, 5.0)	0.0 (0.0, 0.0) *	3.7 (1.0, 8.0)	0.0 (0.0, 0.0) *	0.830	0.907	**<0.001**	0.970
Water, g	2594 (2005, 2900)	2299 (2069, 2844)	2314 (1962, 2645)	2101 (1864, 2682)	0.402	0.466	0.359	0.909
Fibre, g/d	19.5 (16.8, 21.9)	19.5 (16.7, 22.2)	21.9 (19.9, 28.0)	26.8 (17.1, 31.0)	0.068	0.063	0.684	0.841
Cholesterol, mg/d	355 (282, 479)	262 (195, 343)	323 (267, 435)	335 (297, 369)	0.458	0.778	**0.040**	0.115
**Minerals & trace elements**								
Na, mg/d	2571 (2246, 2901)	887 (684, 1151) *	2482 (2309, 2622)	1152 (871, 1615) *	0.943	0.245	**<0.001**	0.122
K, mg/d	2932 (2462, 3513)	2529 (2151, 3257)	3186 (3132, 3605)	2873 (2502, 3236) *	0.259	0.346	**0.001**	0.695
Mg, mg/d	302 (268, 383)	251 (209, 311) *	347 (292, 395)	324 (236, 420)	0.375	0.221	**0.001**	0.514
P, mg/d	1456 (1182, 1562)	1055 (762, 1144) *	1302 (1240, 1448)	999 (801, 1190) *	0.302	0.836	**<0.001**	0.289
Ca, mg/d	1071 (830, 1261)	399 (350, 607) *	1032 (900, 1137)	498 (415, 664) *	0.756	0.435	**<0.001**	**0.044**
Fe, mg/d	11.9 (9.5, 14.0)	9.1 (6.6, 12.1) *	12.3 (10.9, 13.6)	11.3 (9.3, 12.2) *	0.650	0.323	**0.001**	0.525
**Vitamins**								
Vitamin A, µg RE/d	773 (559, 1093)	778 (392, 831)	1281 (870, 1590) #	850 (746, 1414)	**0.022**	**0.031**	0.103	0.400
Vitamin B_1_, mg/d	1.3 (1.1, 1.5)	1.1 (0.8, 1.2) *	1.2 (1, 1.3)	1.1 (0.9, 1.2)	0.239	0.637	**0.001**	0.068
Vitamin B_2_, mg/d	2.0 (1.6, 2.1)	0.9 (0.7, 1.1) *	1.8 (1.7, 2.1)	1.1 (0.9, 1.4) *	0.905	0.471	**<0.001**	0.177
Vitamin B_3_ mg/d	19.7 (15.8, 22.8)	23.5 (16.5, 26.4)	17.6 (14.5, 22.5)	19.0 (14.9, 23.4)	0.488	0.251	0.090	0.396
Vitamin B_5_, mg/d	6.1 (5.5, 7.1)	4.7 (3.9, 6.0) *	5.6 (4.6, 6.5)	4.6 (4.2, 6.2)	0.350	0.696	**0.001**	0.312
Vitamin B_6_, mg/d	1.9 (1.6, 2.3)	2.0 (1.7, 2.3)	1.6 (1.5, 2.3)	2.0 (1.7, 2.3)	0.488	0.514	0.090	0.804
Vitamin B_9_, µg/d	308.9 (260.7, 356.7)	272.3 (219.7, 386.2)	375.0 (329.9, 390.3) #	336.7 (257.8, 441.8)	**0.025**	0.079	0.201	0.704
Vitamin B_12_, µg/d	4.7 (4.0, 8.0)	4.3 (3.2, 4.9) *	5.8 (4.2, 14.2)	9.5 (6.5, 12.5) #	0.302	**0.007**	0.950	0.056
Vitamin C, mg/d	62.7 (45.7, 98.4)	105.1 (51.1, 182.9)	95.2 (81.5, 114.9)	133.0 (98.4, 156.0) *	0.085	0.142	**0.001**	0.993
Vitamin D, µg/d	4.2 (3.4, 5.4)	3.3 (2.2, 3.8)	4.2 (3.6, 5.1)	5.0 (3.1, 6.1)	0.943	0.235	0.159	0.209
Vitamin E, mg/d	9.7 (9.5, 11.7)	13.5 (10.6, 16.3) *	12.0 (10.5, 13.5)	18.1 (14.0, 23.8) *	0.116	0.082	**<0.001**	0.520

Differences in age group at baseline were assessed by a non-paired Wilcoxon signed-rank test (*p*-value < 0.05) (Wilcoxon 1); * Significant difference between periods (diet effect, *p*-value < 0.05) by paired Wilcoxon signed-rank test (Wilcoxon 2); # Significant difference between groups (age effect, *p*-value < 0.05) by non-paired Wilcoxon signed-rank test (Wilcoxon 3); ^a^ *p*-value calculated by Wald Chi-Squared test, statistical significance is indicated in bold when *p*-value < 0.05. Ca: calcium; %E: percent of total energy; Fe: iron; IQR: interquartile range; K: potassium; Mg: magnesium; MUFA: Monounsaturated fatty acids; Na: sodium; OA: older adult men; OB: observation; P: phosphorus; PUFA: Polyunsaturated fatty acids; SC: semi-controlled diet; SFA: Saturated fatty acids; YA: young adult men.

**Table 3:** Fasting clinical parameters (median (IQR)) during observation and semi-controlled diet periods in young and older men.

	YA		OA		*p*-Value (Wilcoxon 1)	*p-*Value (Wald Test) ^a^		
	OB Phase	SC Phase	OB Phase	SC Phase	Baseline OA vs. YA	Age Effect	Diet Effect	Interaction
**Body weight, kg**	80.7 (76.0, 84.4)	79.1 (75.1, 83.6)	73.0 (68.5, 76.4) #	71.4 (67.0, 74.9) #	**0.049**	**0.029**	**<0.001**	0.963
**BMI, kg/m^2^**	25.7 (22.6, 26.3)	25.1 (22.3, 25.9)	24.3 (23.2, 26.8)	23.8 (22.6, 26.9)	0.981	0.990	**<0.001**	0.725
**Biochemical parameters**								
Insulin, pM	40.16 (24.34, 45.39)	26.32 (15.06, 33.93)	22.60 (19.10, 39.00)	21.60 (14.34, 27.43)	0.202	0.251	**0.002**	0.515
Glucose, mM	5.31 (4.95, 5.56)	4.98 (4.87, 5.21)	5.40 (5.20, 5.70)	5.19 (5.03, 5.35)	0.275	0.106	**<0.001**	0.850
Triglycerides, mM	0.99 (0.76, 1.27)	0.67 (0.61, 1.06)	0.90 (0.80, 1.10)	0.93 (0.74, 1.20)	0.734	0.495	0.135	0.091
Total cholesterol, mM	4.43 (4.20, 4.95)	3.98 (3.59, 4.53) *	5.70 (5.30, 6.40) #	5.06 (4.88, 5.84) #	**0.001**	**<0.001**	**<0.001**	0.698
HDL, mM	1.23 (1.11, 1.41)	1.14 (1.01, 1.37)	1.40 (1.30, 1.60)	1.21 (1.15, 1.41)	0.126	0.207	**<0.001**	0.117
LDL, mM	2.71 (2.52, 3.08)	2.28 (2.06, 2.62)	3.60 (3.10, 3.80) #	3.36 (2.79, 3.56) #	**0.001**	**<0.001**	**<0.001**	0.815
Total cholesterol/HDL ratio	1.72 (1.60, 1.79)	1.72 (1.691, 1.857)	1.70 (1.50, 1.80)	1.640 (1.46, 1.86)	0.402	0.271	0.604	0.304
Total cholesterol/LDL ratio	3.75 (3.27, 4.28)	3.31 (3.24, 4.06)	3.90 (3.70, 4.30)	4.132 (3.90, 4.58) #	0.350	**0.045**	0.461	**0.018**
LDL/HDL ratio	2.27 (1.99, 2.57)	1.96 (1.80, 2.33)	2.40 (2.10, 2.70)	2.78 (2.07, 3.10) #	0.375	0.060	0.202	**0.036**
NEFA, mM	0.20 (0.14, 0.23)	0.21 (0.12, 0.26)	0.20 (0.10, 0.20)	0.19 (0.15, 0.23)	0.495	0.907	0.906	0.519
Urea, mM	5.89 (5.44, 6.22)	5.26 (4.89, 6.22)	6.40 (5.70, 7.20)	6.28 (5.09, 7.52)	0.141	0.070	0.109	0.772
Lactate, mM	1.55 (1.37, 1.80)	1.61 (1.41, 1.88)	1.60 (1.30, 1.70)	1.52 (1.43, 1.71)	0.771	0.796	0.554	0.958
**Inflammation parameters**								
CRP, ng/mL	19.36 (16.71, 28.80)	19.327 (12.42, 21.48)	39.20 (24.80, 54.50) #	40.65 (23.20, 69.84) #	**0.038**	**0.002**	0.087	0.087
Adiponectin, ng/mL	8.99 (8.47, 10.71)	9.13 (7.18, 10.67)	7.70 (6.20, 7.90) #	6.29 (5.81, 7.86) #	**0.012**	**0.003**	0.737	0.920

Differences in age group at baseline were assessed by a non-paired Wilcoxon signed-rank test (*p*-value < 0.05) (Wilcoxon 1); * Significant difference between periods (diet effect, *p*-value < 0.05) by paired Wilcoxon signed-rank test (Wilcoxon 2); # Significant difference between groups (age effect, *p*-value < 0.05) by a non-paired Wilcoxon signed-rank test (Wilcoxon 3); ^a^ *p*-value calculated by Wald Chi-Squared test, statistical significance is indicated in bold when *p*-value < 0.05. CRP: C-reactive protein; HDL: high density lipoprotein; IQR: interquartile range; LDL: low density lipoprotein; NEFA: Non-esterified fatty acids; OA: older adult men; YA: younger adult men.

**Table 4:** Fasting serum levels (median (IQR)) of blood metabolites during observation and semi-controlled diet periods in young and older men.

	YA		OA		*p*-Value (Wilcoxon 1)	*p-*Value (Wald Test) ^a^		
	OB Phase	SC Phase	OB Phase (IQR)	SC Phase (IQR)	Baseline OA vs. YA	Age Effect	Diet Effect	Interaction
**Amino acids**								
Total amino acids, A.U.	14.53 (12.64, 18.54)	13.21 (10.59, 16.21)	13.40 (10.32, 18.48)	14.41 (10.65, 16.02)	0.650	0.678	0.203	0.777
Essential amino acids, A.U.	6.60 (6.19, 7.61)	5.70 (4.83, 7.46)	5.79 (5.48, 8.00)	6.93 (4.87, 7.41)	0.793	0.891	0.283	0.849
BCAA, A.U.	2.11 (1.81, 2.55)	2.10 (1.60, 2.31)	2.36 (2.06, 3.00)	2.09 (1.67, 2.49)	0.202	0.498	**0.016**	0.297
Alanine, A.U.	0.97 (0.84, 1.35)	0.92 (0.67, 1.21)	0.95 (0.66, 1.67)	1.10 (0.73, 1.27)	0.943	0.724	0.809	0.600
Asparagine, A.U.	0.45 (0.25, 0.69)	0.41 (0.29, 0.74)	0.62 (0.38, 0.78)	0.65 (0.42, 0.73)	0.259	0.227	0.942	0.844
Aspartic acid, A.U.	0.58 (0.41, 0.81)	0.48 (0.46, 0.68)	0.47 (0.35, 0.68)	0.49 (0.29, 0.72)	0.519	0.431	0.631	0.934
Cysteine, A.U.	0.76 (0.47, 1.30)	0.82 (0.59, 1.35)	0.90 (0.68, 1.20)	0.86 (0.41, 1.28)	0.793	0.918	0.765	0.518
Glutamic acid, A.U.	0.64 (0.57, 0.73)	0.59 (0.46, 0.91)	0.43 (0.36, 0.72)	0.55 (0.31, 0.63)	0.350	0.231	0.479	0.994
Glycine, A.U.	1.20 (0.87, 1.31)	1.05 (0.75, 1.32)	0.86 (0.69, 1.02)	0.92 (0.66, 1.13)	0.169	0.168	0.671	0.615
Isoleucine, A.U.	0.74 (0.58, 0.83)	0.70 (0.58, 0.86)	0.62 (0.58, 1.09)	0.65 (0.52, 0.80)	0.793	0.750	0.194	0.341
Leucine, A.U.	0.61 (0.52, 0.84)	0.55 (0.44, 0.70)	0.84 (0.58, 1.00)	0.65 (0.49, 0.81)	0.169	0.318	**0.005**	0.307
Lysine, A.U.	1.00 (0.81, 1.13)	0.89 (0.71, 1.11)	0.93 (0.64, 1.00)	1.03 (0.90, 1.11)	0.550	0.866	0.497	0.172
Methionine, A.U.	0.85 (0.67, 1.11)	0.78 (0.61, 1.03)	0.77 (0.52, 1.00)	0.87 (0.45, 1.00)	0.402	0.646	0.725	0.424
Phenylalanine, A.U.	0.95 (0.77, 1.05)	0.80 (0.69, 0.99)	0.81 (0.69, 0.98)	0.98 (0.59, 1.06)	0.488	0.839	0.842	0.274
Proline, A.U.	1.18 (0.60, 2.28)	0.82 (0.29, 1.50)	1.02 (0.42, 1.81)	0.94 (0.30, 1.40)	0.375	0.463	0.127	0.846
Serine, A.U.	0.87 (0.50, 1.05)	0.75 (0.44, 1.03)	0.60 (0.48, 1.11)	0.54 (0.50, 0.91)	0.720	0.534	0.327	0.769
Taurine, A.U.	1.36 (1.17, 2.04)	1.39 (1.12, 1.56)	0.99 (0.69, 1.70)	1.36 (0.79, 1.57)	0.155	0.176	0.658	0.584
Threonine, A.U.	0.85 (0.65, 1.05)	0.83 (0.62, 1.08)	0.80 (0.55, 0.96)	0.68 (0.50, 0.89)	0.430	0.267	0.503	0.728
Tryptophan, A.U.	0.86 (0.62, 1.22)	0.80 (0.52, 0.96)	0.87 (0.60, 0.92)	0.63 (0.60, 0.87)	0.458	0.551	0.135	0.522
Tyrosine, A.U.	0.87 (0.70, 0.99)	0.78 (0.63, 0.84)	0.94 (0.83, 0.99)	0.88 (0.79, 0.98)	0.519	0.181	**0.018**	0.507
Valine, A.U.	0.84 (0.74, 0.94)	0.75 (0.65, 0.83)	0.93 (0.89, 1.01)	0.85 (0.67, 0.88) *	0.105	0.253	**0.001**	0.274
**Carbohydrates and derivatives**								
Lactose, A.U.	0.15 (0.13, 0.25)	0.12 (0.10, 0.16) *	0.17 (0.16, 0.23)	0.13 (0.11, 0.17)	0.720	0.517	**<0.001**	0.731
Galactose, A.U.	0.00 (0.00, 0.00)	0.00 (0.00, 0.00)	0.00 (0.00, 0.00)	0.00 (0.00, 0.00)	NA	NA	NA	NA
Galactonate, A.U.	0.08 (0.07, 0.13)	0.07 (0.04, 0.09)	0.14 (0.12, 0.15)	0.08 (0.07, 0.14)	0.054	**0.005**	0.067	0.767
Galactitol, A.U.	0.45 (0.40, 0.57)	0.46 (0.33, 0.57)	0.71 (0.54, 0.77) #	0.72 (0.52, 0.84) #	**0.033**	**0.001**	0.779	0.514
Maltose, A.U.	1.18 (0.94, 1.99)	1.12 (0.63, 1.69)	1.04 (0.64, 1.48)	0.79 (0.65, 1.17)	0.350	0.254	0.070	0.926
**Fatty acids (sums of individual FA)**							
Total fatty acids, mg/L	257.4 (247.1, 296.6)	269.7 (259.9, 302.0)	291.5 (273.4, 328.6) #	301.5 (261.2, 345.8)	**0.025**	0.079	0.626	0.131
SCFA (C4-C10), mg/L	0.0 (0.0, 0.1)	0.1 (0.0, 0.1)	0.1 (0.0, 0.1)	0.1 (0.0, 0.1)	0.756	0.686	0.703	0.987
MCFA (C11-C16), mg/L	77.6 (72.0, 92.4)	78.3 (72.4, 84.3)	91.2 (88.5, 104.5) #	86.0 (72.7, 102.6)	**0.048**	0.068	0.265	0.241
LCFA (>C17), mg/L	159.6 (151.2, 181.0)	174.5 (160.6, 194.3)	180.7 (164.8, 194.7) #	190.2 (159.4, 214.5)	**0.038**	0.167	0.236	0.078
SFA, mg/L	106.0 (99.6, 121.6)	102.0 (96.4, 110.1)	121.1 (117.8, 132.3) #	113.5 (97.6, 134.4)	**0.022**	**0.043**	0.060	0.203
SFA, % of total fatty acids	40.7 (40.1, 41.9)	37.8 (36.1, 38.7) *	41.5 (40.5, 41.7)	38.0 (37.6, 38.7) *	0.616	0.480	**<0.001**	0.889
USFA, mg/L	131.8 (124.0, 150.9)	151.4 (134.8, 172.2) *	152.3 (133.6, 162.5)	160.5 (134.6, 181.7)	0.068	0.202	**0.040**	0.143
MUFA, mg/L	64.6 (50.8, 72.6)	79.6 (69.0, 95.0) *	68.2 (61.7, 82.8)	81.9 (61.9, 100.0)	0.325	0.530	**0.004**	0.201
MUFA, % of total fatty acids	23.4 (21.6, 25.7)	28.0 (26.4, 30.2) *	22.5 (21.6, 26.6)	26.3 (24.4, 31.2)	0.943	0.869	**<0.001**	0.571
PUFA, mg/L	72.5 (67.2, 74.0)	75.3 (64.6, 79.4)	80.0 (75.3, 90.7) #	74.3 (71.4, 80.8)	**0.003**	**0.034**	0.564	0.058
PUFA, % of total fatty acids	27.6 (26.6, 28.0)	26.1 (25.2, 27.8)	27.6 (25.0, 28.1)	26.6 (24.5, 27.1)	0.720	0.616	0.140	0.826
Omega 3 fatty acids, mg/L	10.0 (8.6, 11.6)	11.8 (9.1, 12.7)	16.6 (13.5, 18.9) #	12.4 (12.1, 15.3)	**0.001**	**<0.001**	0.696	**0.047**
Omega 6 fatty acids, mg/L	61.0 (56.8, 66.6)	63.2 (56.4, 70.5)	65.8 (62.9, 69.0)	62.0 (58.0, 67.9)	0.128	0.389	0.752	0.161
BCFA, mg/L	1.4 (1.0, 1.5)	1.2 (0.8, 1.4)	1.8 (1.3, 2.2) #	1.2 (0.9, 1.3) *	**0.022**	0.141	**<0.001**	0.082
TFA, mg/L	4.9 (4.0, 5.6)	4.4 (4.1, 5.1)	5.3 (4.8, 6.5)	4.9 (4.3, 5.7)	0.202	0.189	0.080	0.789
TFA without CLA, mg/L	4.4 (3.8, 4.9)	4.1 (3.8, 4.7)	4.5 (3.9, 5.9)	4.4 (3.7, 5.4)	0.350	0.424	0.206	0.706
CLA, mg/L	0.5 (0.4, 0.6)	0.4 (0.2, 0.5)	0.7 (0.5, 0.9)	0.5 (0.4, 0.6)	0.061	0.096	**0.001**	0.639

Differences in age group at baseline were assessed by a non-paired Wilcoxon signed-rank test (*p*-value < 0.05) (Wilcoxon 1); * Significant difference between periods (diet effect, *p*-value < 0.05) by paired Wilcoxon signed-rank test (Wilcoxon 2); # Significant difference between groups (age effect, *p*-value < 0.05) by Wilcoxon signed-rank test (Wilcoxon 3); ^a^ *p*-value calculated by Wald Chi-Squared test, statistical significance is indicated in bold when *p*-value < 0.05. A.U.: arbitrary unit; BCAA: branched-chain amino acids; BCFA: branched-chain fatty acids; CLA: conjugated linoleic acids; FA: fatty acids; IQR: interquartile range; LCFA: long-chain fatty acids; MCFA: medium-chain fatty acids; MUFA: monounsaturated fatty acids; NA: not available; OA: older adult men; PUFA: polyunsaturated fatty acids; SCFA: short-chain fatty acids; SFA: saturated fatty acids; TFA: *trans* fatty acids; USFA: unsaturated fatty acids; YA; younger adult men.

## Data Availability

The data presented in this study are available on request from the corresponding author.

## Supplementary Materials

The following are available online at https://www.mdpi.com/article/10.3390/nu13061905/s1: Figure S1: Flow diagram of the participants of the study. Figure S2: Principal components analysis (PCA) plot of individual dietary modification during the SC phase. Figure S3: Associations between biochemical parameters and food subgroups that are significantly changed during the SC phase evaluated using Spearman’s correlation. Figure S4. Associations between biochemical parameters that are significantly changed during the SC phase and all food groups evaluated using Spearman’s correlation. Figure S5. Associations between biochemical parameters and nutrients that are significantly changed during the SC phase evaluated using Spearman’s correlation. Table S1. Dietary restriction guidelines for volunteers (original document in French). Table S2. Classification of food groups based on French Agency for Food, Environmental and Occupational Health & Safety (ANSES). Table S3. Fasting free fatty acids in serum (median (IQR)) during observation and semi-controlled diet periods in young and older men.
