# Supplementary material for: Microbiota and Metabolite Modifications after Dietary Exclusion of Dairy Products and Reduced Consumption of Fermented Food in Young and Older Men
Source: Nutrients. 2021 Jun 1;13(6):1905. doi: 10.3390/nu13061905 (PMC8228243; doi:10.3390/nu13061905)
Supplement: Supplementary file 1 [file nutrients-13-01905-s001.zip › nutrients-1202995-supplementary.pdf]

Supplementary Material

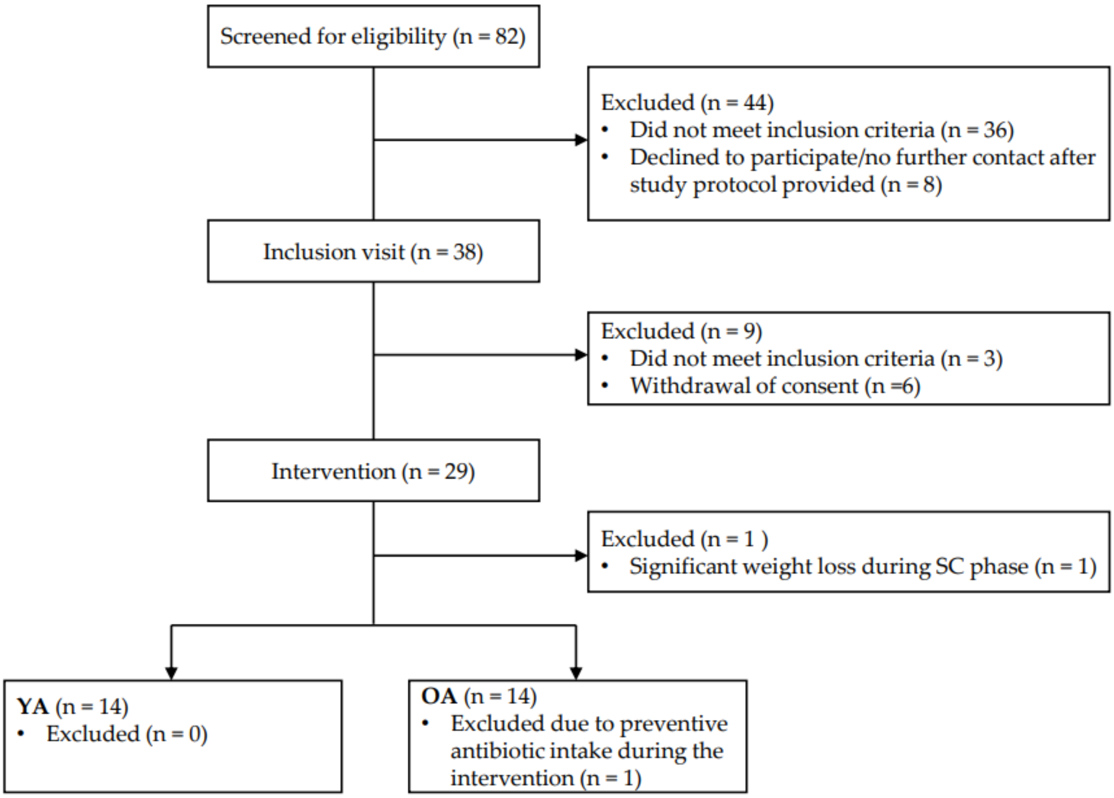

Figure S1. Flow diagram of the participants of the study. OA, older adults; YA, young adults.

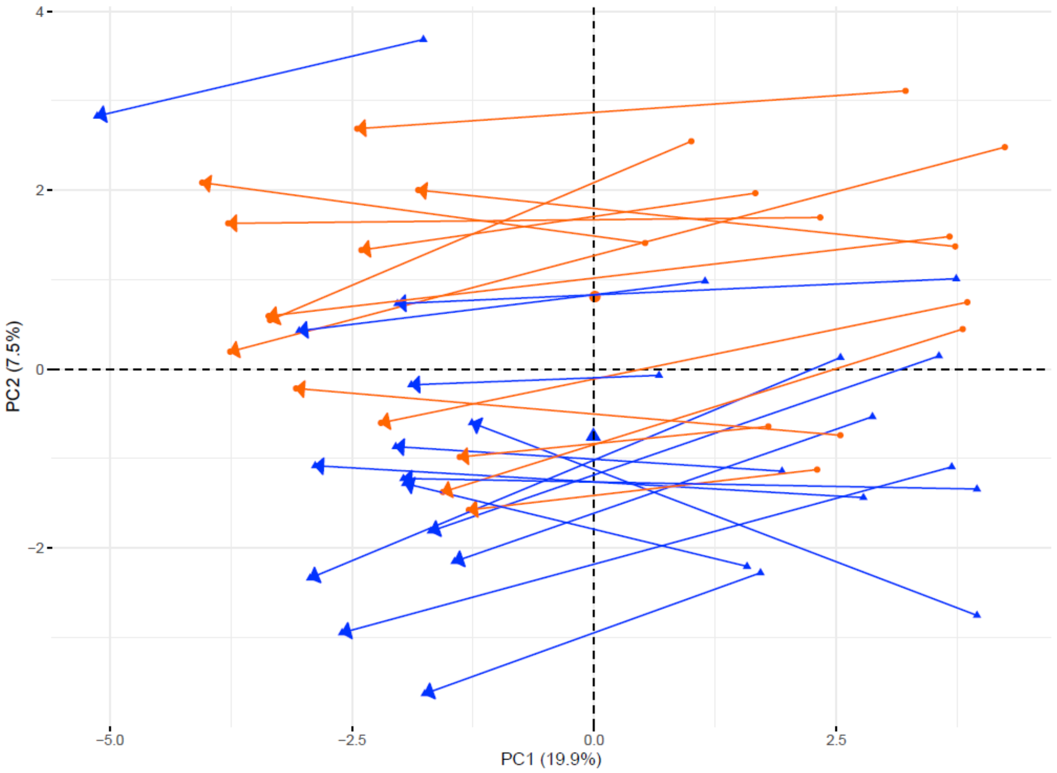

Figure S2. Principal component analysis (PCA) plot of individual dietary modification during the semi-controlled (SC) phase. Young adults (YA): blue arrow; Older adults (OA): orange arrow. PC, principal component.

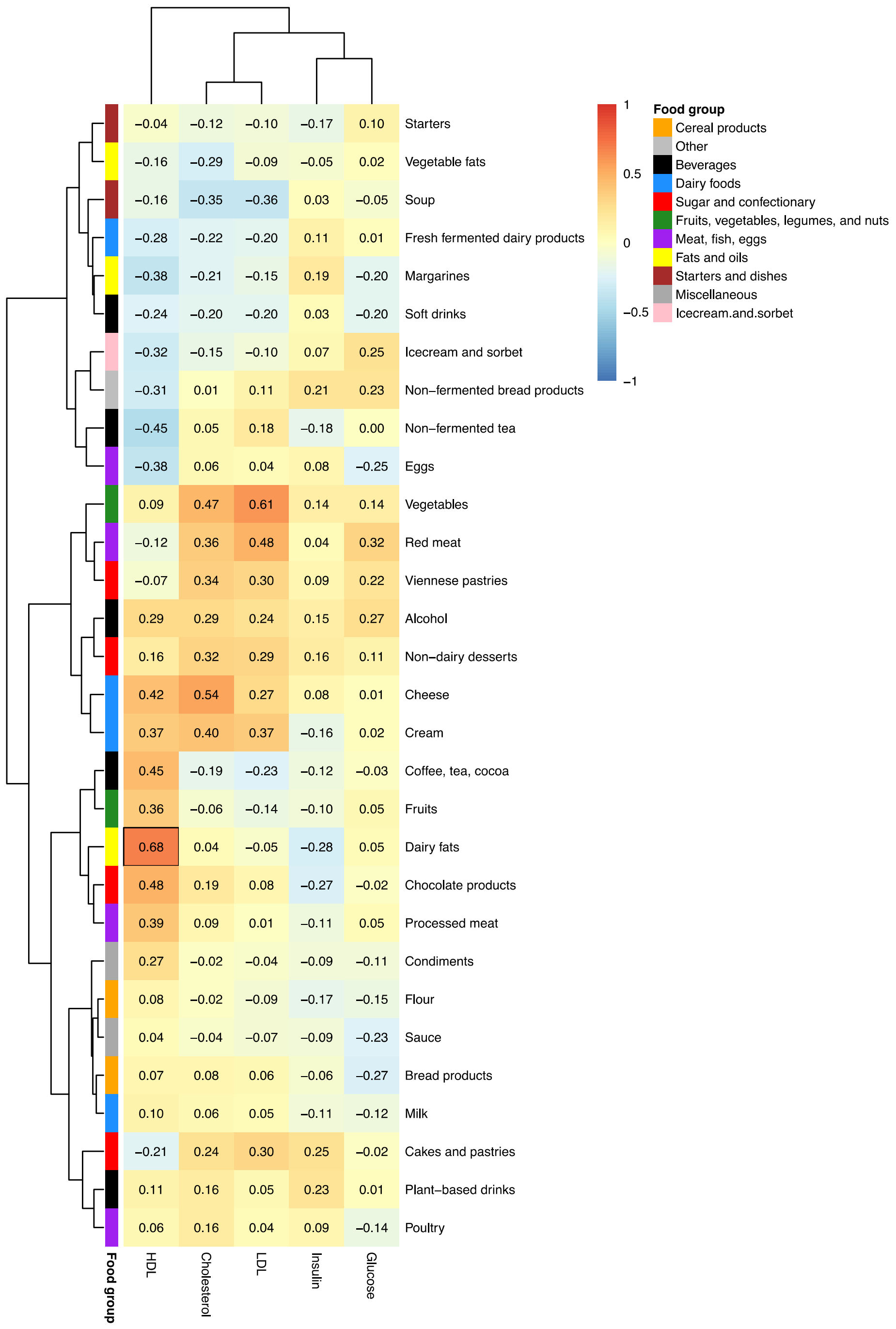

Figure S3. Associations between biochemical parameters and food subgroups that are significantly changed during the SC phase evaluated using Spearman's correlation. Associations are colored and labelled according to Spearman rho, from -1 (negative association) to 1 (positive association). Significant associations are highlighted by a black border (FDR < 0.05). Hierarchical clustering is performed by Ward D2 method using Euclidean distances. Side colors show food group classifications.

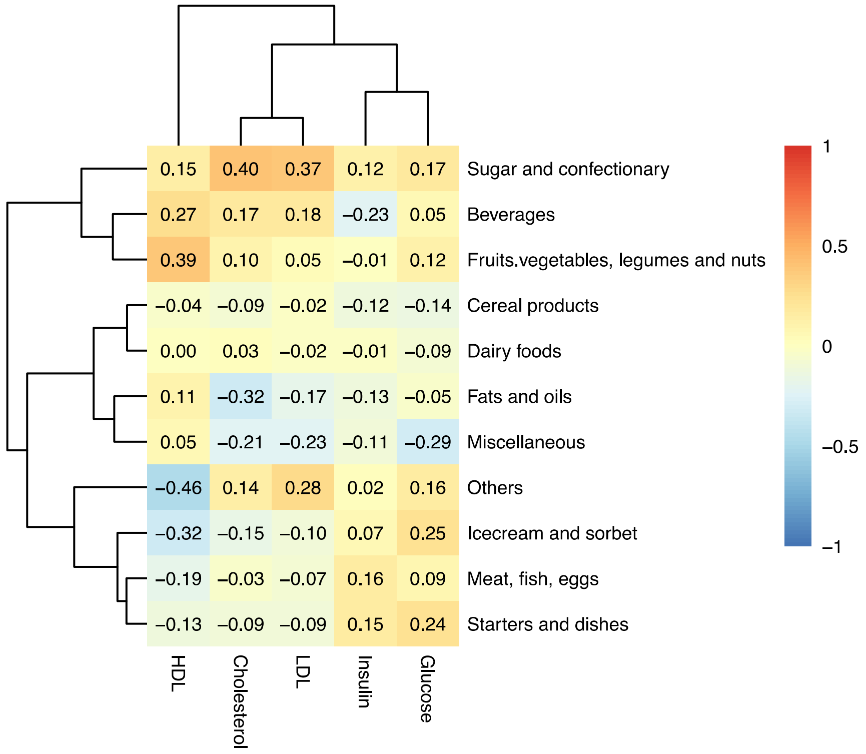

Figure S4. Associations between biochemical parameters that are significantly changed during the SC phase and all food groups evaluated using Spearman's correlation. Associations are colored and labelled according to Spearman rho, from -1 (negative association) to 1 (positive association). Significant associations are highlighted by a black border (FDR < 0.05). Hierarchical clustering is performed by Ward D2 method using Euclidean distances.

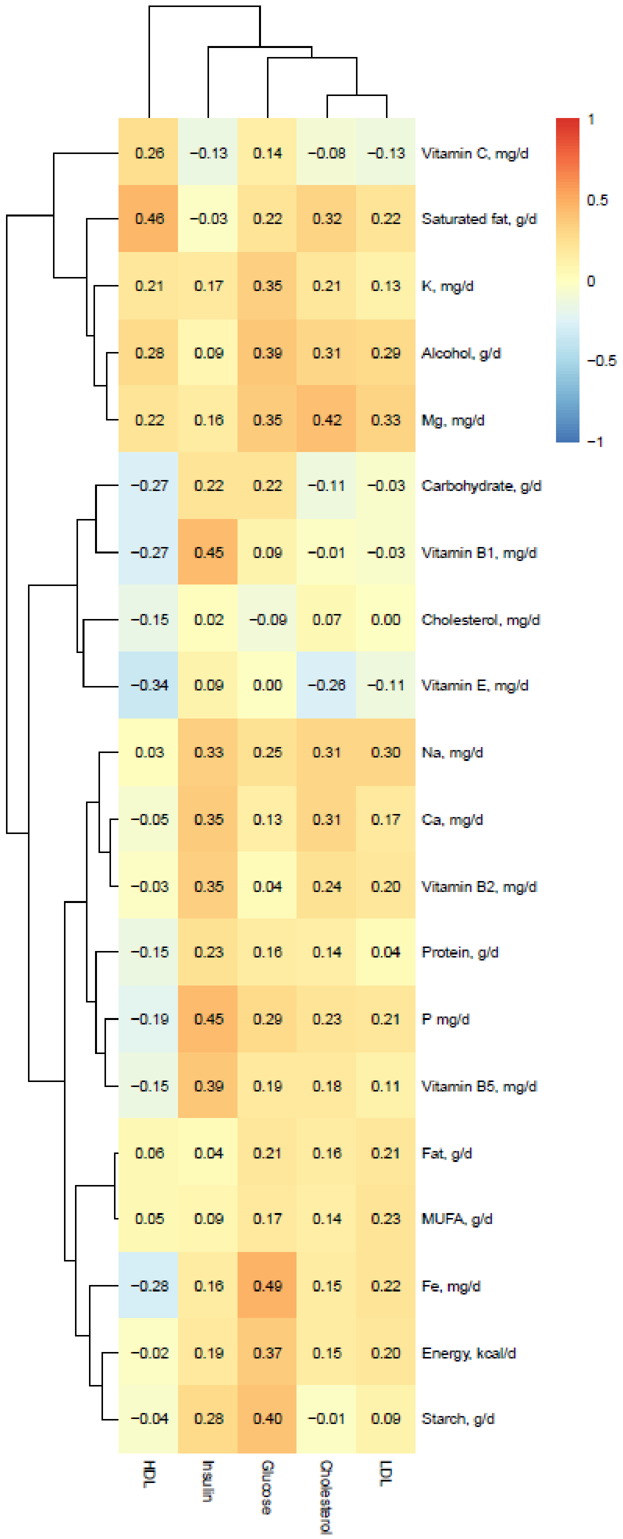

Figure S5. Associations between biochemical parameters and nutrients that are significantly changed by the SC phase (diet effect or interaction), evaluated using Spearman's correlation. Associations are colored and labelled according to Spearman rho, from -1 (negative association) to 1 (positive association). Significant associations are highlighted by a black border (FDR < 0.05). Hierarchical clustering is performed by Ward D2 method using Euclidean distances.

Table S1. Dietary restriction guidelines for the volunteers (original document in French).

Food group	Allowed	Not allowed
Cereal products	Breakfast cereals*<br>Unleavened bread<br>Rice cakes<br>Wheat or corn tortillas, plain Indian bread (or naan)<br>Commercial crackers without yeast * (buckwheat, chestnut, multigrain, quinoa)	**Fermented cereals**:<br>All traditional breads made with baker's yeast, sourdough bread, pastries (croissant, pain au chocolate, braid, brioche ...), pancakes, rusks, crunchy sandwiches (e.g. Cracotte®), toasted breads, Swedish breads<br>**All products with yeast** (exception: yeast extract)
Fruits and vegetables	Fresh, frozen, canned, sterilized fruit and vegetables<br>Juices, purees, compotes, sorbets<br>Soups *<br>Nuts (almonds, hazelnuts, walnuts, cashews, peanuts)	**Fermented vegetables**: pickles, olives, sauerkraut, fermented bamboo, pickled vegetables and other pickled vegetables, lacto-fermented vegetables (soaked in brine), kimchi (fermented cabbage with shallot, peppers, ginger) and lacto-fermented vegetable and plant juices<br>**Fermented fruits**: candied lemons, salted umeboshi plums, fruit kefir (e.g. tibicos)<br>**Dried fruits**
Legumes	Lentils, chickpeas, split peas, red beans, white beans, flageolet beans<br>Fresh and canned sterilized soybeans, firm or silky tofu	Soy sauce (or Shoyu), tamari<br>Soy desserts<br>Fermented soy cheeses (e.g. Sojami®, Sojabio®)<br>Fermented soybeans: tempeh, miso, natto
Starchy food	Pasta, wheat, bulgur, semolina, flour<br>Rice<br>Corn, cornstarch<br>Quinoa<br>Potatoes *	Store-bought preparations and ready meals (potato flakes for mash, potato gratin, shepherd's pie ...) "which contain dairy products "(check labels)
Meat from ruminant animals: veal, beef, lamb, sheep	Fresh or frozen plain meats: all pieces	**Products derived from ruminant meat** (broths, corned beef,<br>merguez) and cured meats (dried meat from Grisons, meat sausages<br>beef or donkey)
Pork	Roasted or pan-fried fillet<br>Roasted, pan-fried, sautéed or grilled Tenderloin / filet mignon<br>Pork ribs<br>Poached, roasted or stewed shoulder or pallet<br>Grilled ribs<br>Grilled or poached fresh breast	**Cured pork**: ribs and ribs for salted snacks, **pork foot, half-salted smoked bacons**<br>**Processed meat (Charcuterie):** raw and dry ham, smoked cooked ham, bacon, speck, dry sausage, salami, chorizo, pepperoni, rillettes, cooked ham, ham, sausages
Other meat	Fresh or frozen plain poultry: chicken, turkey, duck, guinea fowl, quail<br>Rabbit<br>Horse	**Marinated game meat**
Fish and seafood	Fresh, frozen, canned plain fish*	**Salted fish:** sour herring (salted and smoked) and 'roll-mops', anchovies, haddock, salted and dried cod, smoked trout and salmon<br>Gravlax and marinated fish (specifically in vinegar)<br>Fish sauce or nuoc-mâm, caviar, bottarga
Egg	In all their forms: shell, soft-boiled, hard, fried, in a plain omelette<br>Store-bought egg-based preparations and meals that " do not contain dairy products "(look at the labels)	Commercial preparations and ready meals made from eggs and "which contain dairy products "(check labels)
Dairy products		**Milk & fermented milks** (Ribot milk, kefir, koumis, lassi, leben), whey or buttermilk, cheeses, quark, serac, yogurts, white cheeses, faisselles (fromage blanc), fromage frais ('petits suisses')(e.g. Actimel®, Activia®) ...<br>**Smoothies, milkshakes**<br>**Concentrated milk**

Home-made food or ready-to-eat food from stores	Biscuits & cakes (recipes without milk / dairy products, without butter)<br>Commercial preparations "which do not contain dairy products"<br>(check labels)	Biscuits & cakes, home or store "which contain dairy "(check labels)<br>**Dairy desserts:** dessert cream, crème brûlée, custard, cream<br>pastry, dairy desserts & ice cream<br>Pizzas & quiches<br>Industrial sandwiches
Fat	100% vegetable margarines<br>Vegetable oils: olive, sunflower, rapeseed, grape seeds, walnuts, hazelnuts, peanuts, soy	**Dairy fat:** Unsalted, semi-salted, salted butter<br>Crème fraîche, coffee cream, sour cream
Condiments and sauces	Coulis, pulp, pureed tomatoes<br>Vegetable broth<br>Soybean cooking	Wine vinegar, cider vinegar, mustard, homemade or store-bought vinaigrette sauces Mayonnaise, ketchup, tabasco, Viandox® sauce, bouillon of meat, Soy sauce or shoyu, Maggi ® sauce, fish sauce or nuoc-mâm, Worcestershire ® Chutney sauce or sweet and sour sauce<br>Pesto sauce
Drinks	Water (if possible rich in calcium)<br>Fruit juices and herbal teas<br>Green tea & white tea<br>Almond drink *<br>Soy drink *	Black tea, kombuch, coffee, cappuccino, chocolate drinks (e.g. Ovomaltine®)<br>Milk, water & fruit kefirs, cereal drinks (oats, rice, etc.)<br>Energy drinks, regular, low-sugar and sugar-free sodas<br>**All alcoholic drinks:** wine, beer (with or without alcohol), cider, mead, whiskey, vodka
Sugary products	Jam, honey, sugar (maximum 20 g per day, i.e. 4 lumps of sugar n ° 4 or 4 tsp), sour candy.	Milk, cocoa & chocolate spread, milk concentrate, vanilla, caramels (in all its forms), low-fat confectionery in sugar or without sugar (candy, chewing gum)
Divers	Baking powder / sodium bicarbonate	All food supplements<br>Baker's yeast<br>Diet yeast

*Ready-to-eat meals from stores may contain dairy products and / or fermented foods, please check the labels.

Table S2. Classification of food groups based on French Agency for Food, Environmental and Occupational Health & Safety (ANSES).

Food groups	Food subgroups	Food
Beverages	Alcohol	Beers
		Cider
		Cocktails
		Liqueurs
		Wine
	Coffee, tea, and cocoa	Coffee tea cocoa drinks
		Coffee tea cocoa powders
	Juice	Juice
	Non-alcoholic drinks	Homemade drinks
	Plant-based drinks	Plant-based drinks
	Soft drinks	Soft drinks with sugar
		Soft drinks without sugar
	Water	Water
Cereal products	Bread products	Bread
		Special bread
		Toasts rusks
	Flour	Flour starches
	Pasta, rice, and cereals	Cooked pasta semolina
		Cooked rice grains
		Dough
		Raw pasta semolina
		Raw rice grains
Dairy products	Cheese	Cheese
	Cream	Creams
	Dairy fat*	Butter
	Fresh fermented dairy products	Fresh dairy desserts
		Cultured soft cheese
		Yogurt
	Milk	Milk
	Other dairy desserts	Canned dairy desserts
		Concentrated or powdered milk
Fats and oils	Animal fat	Other animal fats
	Margarines	Margarines
	Vegetable fats	Vegetable oils
Fruits, vegetables, legumes, and nuts	Fruits	Canned fruits
		Compotes
		Dried freeze dried fruits
		Fruits jams
		Other processed fruit products
		Raw fruits
	Nuts	Nuts
	Potatoes	Root vegetables
	Vegetables	Cooked vegetables
		Dried dehydrated vegetables
		Raw vegetables
		Salad
Ice cream and sorbet	Ice cream and sorbet	Ice cream sorbet
Meat, fish, eggs	Eggs	Egg derivatives
	Fish and seafood	Canned fish products
		Cooked fish
		Fried fish
		Other fish based products
		Raw fish
		Sea food
		Smoked fish
	Offal**	Offal
	Poultry**	Poultry
	Processed meat	Cooked ham

		Dried raw ham
		Dry sausages
		Dumplings
		Other special cured meats
		Pâté terrine
		Rillettes
		Sausages
	Red meat**	Meat
Miscellaneous	Condiments	Condiments
	Cooking aids	Culinary aids
	Ingredients	Chemical yeast bicarbonate
	Sauce	Salted sauces condiments
		Savory sauces
		Sweet sauces
	Spices	Dried spices
		Fresh spices
		Spices
Others ***	Alternative cheese	Cheese alternatives
	Non-dairy desserts	Soy dessert
	Non-fermented bread products	Non fermented and non-dairy bread
	Non-fermented sweet biscuits	Non fermented and non-dairy sugary biscuits
	Non-fermented tea	Green tea infusion
Starters and dishes	Cereal composite dish	Cereal pasta dishes
		Vegetable legume dishes
	Fish composite dish	Fish dishes without garnish
		Meat poultry dish with starchy foods
	Meat composite dish	Meat poultry dish with vegetables
		Meat poultry dishes without garnish
	Pizza	Pizza pancakes savory tarts
	Sandwiches	Sandwiches
	Soup	Soups
	Starters	Puff pastry savory cakes
		Savory biscuits
	Vegetable composite dish	Fish dish with starchy foods
Sugar and confectionary	Breakfast cereals	Crunchy muesli
		Filled breakfast cereals
		Healthy breakfast cereals
		Muesli
		Not filled chocolate breakfast cereals
		Other breakfast cereals
		Rich fiber breakfast cereals
		Sugary honey caramel breakfast cereals
	Cakes and pastries	Cakes pastries
	Cereal bars	Cereal bars
	Chocolate products	Chocolate products
	Jam	Jam
	Non-chocolate confectionery	Non chocolate confectionery
	Non-dairy desserts ****	Fresh non-dairy desserts
	Sugars and honey	Sugar honey syrup
	Sweet biscuits	Sweet biscuits
	Viennese pastries	Brioche

*The food composition table CIQUAL® classifies dairy fats under “fats and oils”. Due to the specificities of this study, dairy fats are classified under “dairy products”. **CIQUAL® separates cooked and raw meat. This study classifies by the type of meat. ***These groups are added in the diet to substitute the exclusion of dairy and fermented food. ***CIQUAL® includes this dairy substitute in “dairy products”; in this study this product is separated to allow an assessment of dairy food intake.

Table S3. Fasting free fatty acids in serum (median (IQR)) during observation and semi-controlled diet periods in young and older men.

Free fatty acids (mg/L)	YA		OA		*P*-value (Wilcoxon 1)	*P*-value (Wald test) [a]		
	OB phase (V1)	SC phase (V3)	OB phase (V1)	SC phase (V3)	Baseline OA vs YA	Age effect	Diet effect	Interaction
**Saturated fatty acids (SFA)**								
Butanoic acid	0.006 (0.004, 0.010)	0.010 (0.008, 0.013)	0.010 (0.005, 0.015)	0.008 (0.005, 0.012)	0.259	0.488	0.449	0.190
Pentanoic acid	0.006 (0.003, 0.009)	0.007 (0.005, 0.010)	0.005 (0.004, 0.010)	0.008 (0.003, 0.011)	0.583	0.939	0.645	0.445
Hexanoic acid	0.005 (0.004, 0.008)	0.007 (0.005, 0.009)	0.007 (0.006, 0.014)	0.009 (0.004, 0.012)	0.185	0.404	0.928	0.240
Heptanoic acid	0.007 (0.005, 0.011)	0.007 (0.004, 0.008)	0.008 (0.004, 0.011)	0.009 (0.005, 0.014)	0.905	0.524	0.816	0.276
Octanoic acid	0.006 (0.004, 0.009)	0.005 (0.004, 0.007)	0.006 (0.004, 0.008)	0.008 (0.004, 0.011)	0.756	0.306	0.895	0.328
Nonanoic acid	0.007 (0.004, 0.009)	0.005 (0.003, 0.009)	0.005 (0.003, 0.007)	0.009 (0.003, 0.011)	0.325	0.868	0.675	0.096
Decanoic acid	0.006 (0.003, 0.007)	0.004 (0.003, 0.008)	0.005 (0.003, 0.010)	0.006 (0.004, 0.010)	1.000	0.506	0.766	0.441
Dodecanoic acid	0.403 (0.357, 0.621)	0.510 (0.311, 0.728)	0.543 (0.330, 0.694)	0.432 (0.296, 0.610)	0.583	0.961	0.733	0.286
11-Methyldodecanoic acid (C13 iso)	0.004 (0.003, 0.006)	0.003 (0.003, 0.005)	0.003 (0.002, 0.005)	0.004 (0.003, 0.005)	0.302	0.877	0.580	0.153
12-Methyltridecanoic acid (C14 iso)	0.004 (0.003, 0.007)	0.006 (0.003, 0.008)	0.004 (0.003, 0.006)	0.005 (0.003, 0.009)	0.943	0.871	0.724	0.688
Tetradecanoic acid	2.708 (2.509, 3.199)	2.928 (2.061, 3.631)	3.330 (2.613, 4.482)	2.560 (2.106, 3.995)	0.259	0.536	0.131	0.223
13-Methyltetradecanoic acid (C15 iso)	0.111 (0.103, 0.128)	0.106 (0.082, 0.174)	0.153 (0.124, 0.177) #	0.113 (0.107, 0.205)	**0.019**	**0.029**	0.478	0.605
12-Methyltetradecanoic acid (C15 aiso)	0.292 (0.230, 0.336)	0.285 (0.202, 0.359)	0.356 (0.273, 0.422)	0.283 (0.250, 0.309) *	0.061	0.438	**0.034**	0.079
Pentadecanoic acid	0.695 (0.618, 0.794)	0.635 (0.547, 0.788)	0.965 (0.760, 1.105) #	0.744 (0.653, 0.930) *	**0.002**	**0.005**	**0.030**	0.152
14-Methylpentadecanoic acid (C16 iso)	0.244 (0.214, 0.282)	0.211 (0.137, 0.248)	0.270 (0.242, 0.312)	0.197 (0.163, 0.227) *	0.116	0.642	**< 0.001**	**0.048**
Hexadecanoic acid	68.312 (63.923, 79.961)	67.890 (62.313, 72.855)	80.066 (77.365, 88.067) #	76.445 (63.816, 90.896)	**0.033**	**0.047**	0.165	0.207
15-Methylhexadecanoic acid (C17 iso)	0.423 (0.337, 0.509)	0.329 (0.246, 0.473)	0.575 (0.505, 0.696) #	0.364 (0.298, 0.442) *	**0.011**	0.064	**< 0.001**	**0.038**
14-Methylhexadecanoic acid (C17 aiso)	0.181 (0.068, 0.216)	0.100 (0.064, 0.168)	0.214 (0.059, 0.367)	0.099 (0.083, 0.177)	0.259	0.334	**0.045**	0.764
Heptadecanoic acid	0.990 (0.945, 1.159)	1.006 (0.855, 1.309)	1.290 (1.206, 1.509) #	1.165 (0.981, 1.380)	**0.001**	**0.001**	0.289	0.132
16-Methylheptadecanoic acid (C18 iso)	0.008 (0.004, 0.143)	0.006 (0.004, 0.016)	0.018 (0.007, 0.187)	0.013 (0.005, 0.025)	0.155	0.103	0.254	0.787
Octadecanoic acid	30.675 (27.271, 31.758)	27.226 (26.156, 31.366)	32.653 (32.311, 39.536) #	30.009 (26.725, 33.140) *	**0.011**	**0.025**	**0.018**	0.190
Nonadecanoic acid	0.316 (0.219, 0.379)	0.342 (0.253, 0.390)	0.253 (0.175, 0.330)	0.239 (0.140, 0.349)	0.220	0.121	0.641	0.721
Icosanoic acid	0.215 (0.184, 0.250)	0.267 (0.206, 0.300)	0.216 (0.187, 0.253)	0.256 (0.174, 0.277)	0.905	0.456	**0.041**	0.301
Docosanoic acid	0.341 (0.262, 0.390)	0.193 (0.174, 0.386)	0.356 (0.273, 0.424)	0.248 (0.220, 0.273) *	0.830	0.851	**< 0.001**	0.674
**Unsaturated fatty acids (USFA)**								
Mono-unsaturated fatty acids (MUFA)								
Dec-9-enoic acid	0.005 (0.005, 0.009)	0.005 (0.004, 0.006)	0.005 (0.004, 0.008)	0.006 (0.003, 0.007)	0.905	0.797	0.170	0.480
Dodecenoic acid (unknown)	0.004 (0.003, 0.006)	0.004 (0.003, 0.005)	0.004 (0.003, 0.005)	0.005 (0.003, 0.007)	0.943	0.604	0.712	0.616
9E-tetradecenoic acid	0.005 (0.004, 0.010)	0.004 (0.003, 0.009)	0.005 (0.004, 0.006)	0.005 (0.003, 0.010)	0.981	0.836	0.406	0.795
(Z)-Tetradec-9-enoic acid	0.207 (0.166, 0.294)	0.243 (0.168, 0.343)	0.248 (0.224, 0.384)	0.212 (0.177, 0.395)	0.350	0.486	0.641	0.408
(E)-hexadec-9-enoic acid	0.126 (0.109, 0.172)	0.109 (0.074, 0.167)	0.180 (0.136, 0.269)	0.141 (0.112, 0.175)	0.141	0.090	0.183	0.873
Hexadecenoic acid (unknown)	0.619 (0.522, 0.798)	0.822 (0.677, 0.927) *	0.745 (0.587, 0.954)	0.886 (0.714, 1.032)	0.325	0.171	**< 0.001**	0.600
(Z)-Hexadec-9-enoic acid	3.711 (2.959, 4.483)	4.648 (3.011, 5.465)	4.757 (3.586, 5.933)	4.511 (4.190, 6.176)	0.185	0.236	0.142	0.359
Hexadecenoic acid (unknown 2)	0.079 (0.059, 0.094)	0.079 (0.038, 0.093)	0.076 (0.058, 0.113)	0.088 (0.048, 0.116)	0.583	0.298	0.849	0.678
(Z)-Heptadec-10-enoic acid	1.373 (0.976, 1.781)	1.501 (1.082, 2.066)	1.446 (1.064, 1.559)	1.509 (0.729, 1.720)	0.616	0.531	**0.008**	0.974

Free fatty acids (mg/L)	YA		OA		*P*-value (Wilcoxon 1)	*P*-value (Wald test) [a]		
	OB phase (V1)	SC phase (V3)	OB phase (V1)	SC phase (V3)	Baseline OA vs YA	Age effect	Diet effect	Interaction
(E)-Octadec-6-enoic acid + (E)-Octadec-9-enoic acid	1.743 (1.450, 2.258)	1.847 (1.337, 2.423)	1.716 (1.533, 2.495)	1.668 (1.549, 2.823)	0.867	0.934	0.495	0.883
(E)-Octadec-10-enoic acid + (E)-Octadec-11-enoic acid	1.139 (1.039, 1.369)	1.046 (0.904, 1.225)	1.287 (1.106, 1.519)	1.106 (0.967, 1.262)	0.202	0.170	**0.009**	0.668
(E)-Octadec-12-enoic acid	0.479 (0.445, 0.544)	0.498 (0.426, 0.561)	0.509 (0.469, 0.540)	0.481 (0.441, 0.563)	0.616	0.706	0.776	0.717
(E)-Octadec-13-enoic acid + (Z)-Octadec-6-enoic acid + (Z)-Octadec-7-enoic acid + octadecenoic acid (unknown)	0.521 (0.467, 0.628)	0.412 (0.355, 0.494) *	0.649 (0.537, 0.732)	0.452 (0.376, 0.545) *	0.076	0.077	**< 0.001**	0.565
(Z)-Octadec-9-enoic acid	47.868 (36.725, 55.919)	62.312 (52.540, 73.244) *	51.090 (44.092, 61.618)	63.823 (45.274, 78.196)	0.402	0.658	**0.001**	0.182
(Z)-Octadec-11-enoic acid	3.608 (3.038, 4.615)	4.841 (4.125, 5.285) *	4.492 (3.993, 5.094)	5.576 (4.218, 5.801)	0.068	0.070	**0.001**	0.391
(Z)-Octadec-12-enoic acid	0.532 (0.456, 0.572)	0.407 (0.309, 0.500)	0.479 (0.423, 0.552)	0.430 (0.337, 0.551)	0.756	0.915	**0.024**	0.505
(Z)-Octadec-13-enoic acid	0.169 (0.151, 0.194)	0.171 (0.148, 0.207)	0.188 (0.165, 0.199)	0.166 (0.151, 0.195)	0.239	0.609	0.215	0.228
Octadecenoic acid (unknown)	0.137 (0.061, 0.243)	0.151 (0.073, 0.325)	0.218 (0.123, 0.520)	0.215 (0.100, 0.500)	0.169	0.179	0.928	0.423
(Z)-Octadec-15-enoic acid	0.071 (0.056, 0.084)	0.065 (0.048, 0.076)	0.061 (0.056, 0.121)	0.056 (0.041, 0.073)	0.943	0.833	0.095	0.726
(E)-Icos-11-enoic acid + (Z)-Icos-5-enoic acid	0.077 (0.054, 0.085)	0.083 (0.065, 0.103)	0.089 (0.059, 0.125)	0.088 (0.057, 0.108)	0.169	0.422	0.580	0.130
(Z)-Icos-8-enoic acid + (Z)-Icos-9-enoic acid	0.205 (0.073, 0.332)	0.105 (0.059, 0.312)	0.188 (0.102, 0.331)	0.258 (0.085, 0.313)	0.793	0.537	0.451	0.547
(Z)-Icos-11-enoic acid	0.661 (0.597, 0.978)	1.015 (0.845, 1.182) *	0.949 (0.690, 1.028)	1.074 (0.967, 1.116)	0.141	0.350	**0.011**	0.351
Poly-unsaturated fatty acids (PUFA)								
(9Z, 11Z)-Octadeca-9,11-dienoic acid	0.020 (0.016, 0.026)	0.020 (0.014, 0.024)	0.020 (0.013, 0.033)	0.024 (0.016, 0.050)	0.830	0.457	0.861	0.490
(9Z,12Z)-Octadeca-9,12-dienoic acid	39.974 (37.295, 43.768)	43.414 (38.812, 46.829)	42.929 (41.971, 47.338)	42.728 (36.359, 47.244)	0.169	0.522	0.826	0.176
(9Z,11E)-Octadeca-9,11-dienoic acid + unknown	0.399 (0.306, 0.503)	0.325 (0.185, 0.414)	0.597 (0.421, 0.718) #	0.338 (0.281, 0.496) *	**0.033**	0.062	**< 0.001**	0.348
(10E,12Z)-Octadeca-10,12-dienoic acid	0.025 (0.012, 0.039)	0.017 (0.013, 0.032)	0.025 (0.015, 0.097)	0.015 (0.012, 0.073)	0.550	0.701	0.140	0.500
(9E,11E)-Octadeca-9,11-dienoic acid	0.014 (0.007, 0.016)	0.014 (0.010, 0.018)	0.017 (0.013, 0.021)	0.017 (0.011, 0.022)	0.202	0.135	0.735	0.714
(9E,12E)-Octadeca-9,12-dienoic acid	0.040 (0.032, 0.072)	0.059 (0.039, 0.098)	0.047 (0.039, 0.091)	0.052 (0.041, 0.114)	0.616	0.836	0.158	0.488
((9E,12Z)-Octadeca-9,12-dienoic acid) + unknown	0.144 (0.095, 0.174)	0.109 (0.069, 0.159)	0.139 (0.102, 0.192)	0.124 (0.113, 0.160)	0.519	0.439	0.136	0.923
Octadecadienoic acid (unknown)	0.137 (0.061, 0.243)	0.151 (0.073, 0.325)	0.218 (0.123, 0.520)	0.215 (0.100, 0.500)	0.169	0.179	0.928	0.423
Octadecadienoic acid (unknown)	0.213 (0.168, 0.281)	0.226 (0.177, 0.294)	0.252 (0.211, 0.271)	0.231 (0.202, 0.310)	0.128	0.241	0.837	0.397
Octadecadienoic acid (unknown)	0.087 (0.071, 0.112)	0.079 (0.053, 0.094)	0.103 (0.071, 0.140)	0.080 (0.071, 0.109)	0.430	0.314	0.067	0.953
(6Z,9Z,12Z)-Octadeca-6,9,12-trienoic acid	0.512 (0.469, 0.595)	0.469 (0.414, 0.511)	0.473 (0.409, 0.587)	0.453 (0.307, 0.529)	0.458	0.359	**0.024**	0.964
(9Z,12Z,15Z)-Octadeca-9,12,15-trienoic acid	1.349 (1.066, 2.015)	1.802 (1.596, 2.110)	1.329 (1.304, 2.692)	1.853 (1.356, 2.338)	0.265	0.423	**0.035**	0.468
(6Z,9Z,12Z,15Z)-Octadeca-6,9,12,15-tetraenoic acid	0.163 (0.108, 0.184)	0.134 (0.098, 0.165)	0.169 (0.023, 0.202)	0.153 (0.042, 0.182)	0.943	0.984	0.086	0.813
(11Z,14Z)-Icosa-11,14-dienoic acid	0.518 (0.277, 0.622)	0.524 (0.361, 0.597)	0.518 (0.214, 0.748)	0.427 (0.197, 0.718)	0.550	0.984	0.876	0.183
(8Z,11Z,14Z)-Icosa-8,11,14-trienoic acid	3.290 (2.967, 3.435)	2.799 (2.134, 3.301)	3.296 (2.624, 4.081)	2.285 (1.935, 2.898) *	0.756	0.374	**< 0.001**	0.606
(11Z,14Z,17Z)-Icosa-11,14,17-trienoic acid	0.065 (0.050, 0.085)	0.072 (0.065, 0.094)	0.089 (0.062, 0.121)	0.085 (0.071, 0.101)	0.128	0.084	0.377	0.527

Free fatty acids (mg/L)	YA		OA		*P*-value (Wilcoxon 1)	*P*-value (Wald test) [a]		
	OB phase (V1)	SC phase (V3)	OB phase (V1)	SC phase (V3)	Baseline OA vs YA	Age effect	Diet effect	Interaction
(5Z,8Z,11Z,14Z)-Icosa-5,8,11,14-tetraenoic acid	14.182 (12.579, 17.122)	14.929 (14.019, 16.649)	16.792 (14.209, 18.547)	14.777 (14.636, 16.639)	0.141	0.464	0.562	0.081
(8Z,11Z,14Z,17Z)-Icosa-8,11,14,17-tetraenoic acid	0.198 (0.148, 0.250)	0.137 (0.101, 0.174) *	0.209 (0.180, 0.269)	0.115 (0.098, 0.160) *	0.458	0.800	**< 0.001**	0.272
(5Z,8Z,11Z,14Z,17Z)-Icosa-5,8,11,14,17-pentaenoic acid (EPA)	1.790 (1.327, 2.851)	1.950 (1.371, 2.657)	2.586 (2.412, 4.116) #	1.967 (1.721, 2.356) *	**0.033**	0.084	0.067	0.091
(7Z,10Z,13Z,16Z,19Z)-Docosa-7,10,13,16,19-pentaenoic acid (DPA)	1.412 (1.219, 1.516)	1.247 (1.085, 1.441)	1.736 (1.526, 2.089) #	1.344 (1.181, 1.665) *	**0.004**	**0.008**	**0.015**	0.192
(4Z,7Z,10Z,13Z,16Z,19Z)-docosa-4,7,10,13,16,19-hexaenoic acid (DHA)	4.848 (4.236, 5.554)	5.851 (4.795, 6.610)	7.778 (6.595, 9.296) #	7.226 (6.876, 7.607) #	**< 0.001**	**< 0.001**	0.506	0.201

Differences in age group at baseline were assessed by a non-paired Wilcoxon signed-rank test (*P*-value < 0.05) (Wilcoxon 1); * Significant difference between periods (diet effect, *P*-value < 0.05) by paired Wilcoxon signed-rank test (Wilcoxon 2); # Significant difference between groups (age effect, *P*-value < 0.05) by Wilcoxon signed-rank test (Wilcoxon 3); [a] *P*-value calculated by Wald Chi-Squared Test, statistical significance is indicated in bold when *P*-value < 0.05.

EPA: Eicosapentaenoic acid; DHA: Docosahexaenoic acid; DPA: Docosapentaenoic acid; IQR: interquartile range; MUFA: monounsaturated fatty acids; OA: older adult men; PUFA: polyunsaturated fatty acids; SFA: saturated fatty acids; USFA: unsaturated fatty acids; YA; younger adult men.
